# Palmitoylation of Metazoan Carotenoid Oxygenases

**DOI:** 10.3390/molecules25081942

**Published:** 2020-04-22

**Authors:** Sheetal Uppal, Igor B. Rogozin, T.Michael Redmond, Eugenia Poliakov

**Affiliations:** 1 Laboratory of Retinal Cell & Molecular Biology, National Eye Institute, National Institutes of Health, Bethesda, MD 20892, USA; sheetal.uppal2@nih.gov (S.U.); redmondd@helix.nih.gov (T.M.R.); 2 National Center for Biotechnology Information, National Library of Medicine, National Institutes of Health, Bethesda, MD 20894, USA; rogozin@ncbi.nlm.nih.gov

**Keywords:** palmitoylation, palmitoylation motif, phylogenetics, carotenoid oxygenases, carotenoids

## Abstract

Abundant in nature, carotenoids are a class of fat-soluble pigments with a polyene tetraterpenoid structure. They possess antioxidant properties and their consumption leads to certain health benefits in humans. Carotenoid cleavage oxygenases (CCOs) are a superfamily of enzymes which oxidatively cleave carotenoids and they are present in all kingdoms of life. Complexity of CCO evolution is high. For example, in this study we serendipitously found a new family of eukaryotic CCOs, the apocarotenoid oxygenase-like (ACOL) family. This family has several members in animal genomes and lacks the animal-specific amino acid motif PDPCK. This motif is likely to be associated with palmitoylation of some animal CCOs. We recently demonstrated that two mammalian members of the carotenoid oxygenase family retinal pigment epithelial-specific 65 kDa protein (RPE65) and beta-carotene oxygenase 2 (BCO2) are palmitoylated proteins. Here we used the acyl-resin-assisted capture (acyl-RAC) method to demonstrate protein palmitoylation and immunochemistry to localize mouse BCO2 (mBCO2) in COS7 cell line in the absence and presence of its substrate β-carotene. We demonstrate that mBCO2 palmitoylation depends on the evolutionarily conserved motif PDPCK and that metazoan family members lacking the motif (Lancelet beta-carotene oxygenase-like protein (BCOL) and Acropora ACOL) are not palmitoylated. Additionally, we observed that the palmitoylation status of mBCO2 and its membrane association depend on the presence of its substrate β-carotene. Based on our results we conclude that most metazoan carotenoid oxygenases retain the evolutionarily conserved palmitoylation PDPCK motif to target proteins to internal membranes depending on substrate status. Exceptions are in the secreted BCOL subfamily and the strictly cytosolic ancient ACOL subfamily of carotenoid oxygenases.

## 1. Introduction

Carotenoids are a class of naturally occurring pigments with antioxidant properties [1,2,3]. β-Carotene and certain other carotenoids such as β-cryptoxanthin have provitamin A activity, and the hydroxylated carotenoid xanthophylls lutein and zeaxanthin have been shown to protect the retina of the eye against oxidative stress [4]. Additionally, carotenoids can be oxidatively cleaved by various carotenoid cleavage oxygenases (CCOs) to generate various regulatory molecules such as hormones in plants (abscisic acid by 9-*cis*-epoxycarotenoid dioxygenase (NCED) subfamily [5,6] or carlactone (carotenoid cleavage dioxygenases 7 and 8 (CCD7, CCD8) [7]), vitamin A (mammalian beta-carotene oxygenase 1 (BCO1) [8] and insect carotenoid isomerooxygenase (NinaB) [9,10]) or retinoid X receptor (RXR)-regulating apocarotenoids (mammalian BCO2) [11]. Carotenoid oxygenases are present in all kingdoms of life and have only been rarely lost in animal genomes [12]. They contain the same basic structural fold of a seven-bladed β-propeller and iron cofactor coordinated by four conserved histidines and three second shell glutamic acid residues [13,14,15,16]. Mutation of any of the conserved histidines leads to total loss of activity for several mammalian carotenoid oxygenases [17,18]. Carotenoid oxygenases act on hydrophobic substrates and their interaction with membrane and availability of substrate from membrane or lipid droplets is not completely known. Most metazoan carotenoid oxygenases contain the conserved PDPCK motif [12,19]. RPE65 retinoid isomerase is an enzyme which plays a crucial role in the regeneration of 11-*cis*-retinal (visual chromophore of rhodopsin) in the visual cycle of the retina [18,20,21]. It is a divergent and atypical CCO in that it neither uses carotenoids as a substrate, nor is it an oxygenase. The C112 cysteine residue located in the PDPCK motif of RPE65 protein is S-palmitoylated and palmitoylation at C112 is responsible for membrane interaction of RPE65. Mutation of C112 to any other residue leads to a complete loss of RPE65 activity [13,22,23]. In addition, the palmitoylation level of RPE65 is modulated by the presence of active lecithin:retinol acyltransferase (LRAT), the obligate enzyme that produces RPE65′s all-*trans*-retinyl ester substrate, suggesting that palmitoylation also plays a functional or catalytic role [23]. Because of the conserved nature of the PDPCK motif in the animal BCO/RPE65 clades of the carotenoid oxygenase superfamily, we postulated that the presence of this motif in any protein belonging to the family would predict palmitoylation of the protein. Recently, we confirmed our hypothesis by demonstrating that mouse BCO2 is also a palmitoylated protein [19]. In this paper, we demonstrate that PDPCK is a palmitoylation motif of BCO2 and that, similar to RPE65, BCO2 palmitoylation depends on the presence of substrate. Our immunofluorescence results confirm the mitochondrial localization of BCO2 protein as shown earlier [24]. In addition to this, we further monitor the changes in subcellular localization upon treatment with substrate [24,25]. Significantly, we also show that mutation of the cysteine residue (C111) in the PDPCK motif of BCO2 protein abolishes palmitoylation of BCO2. Previously, phylogenetic analysis of BCO/RPE65 family has demonstrated the presence of a BCO-like (BCOL) subfamily, where the PDPCK motif is absent. Now, also, we have characterized a new subfamily of ACOL proteins that are unlikely to be due to horizontal gene transfer (HGT) and where the PDPCK motif is also absent. Finally, we have characterized the palmitoylation status of the BCOLg and ACOLa proteins. 

## 2. Results

### 2.1. Palmitoylation of Mouse BCO2 (mBCO2) (without Tag and with Tag) in the Absence and Presence of Its Substrate

**Figure 1:**
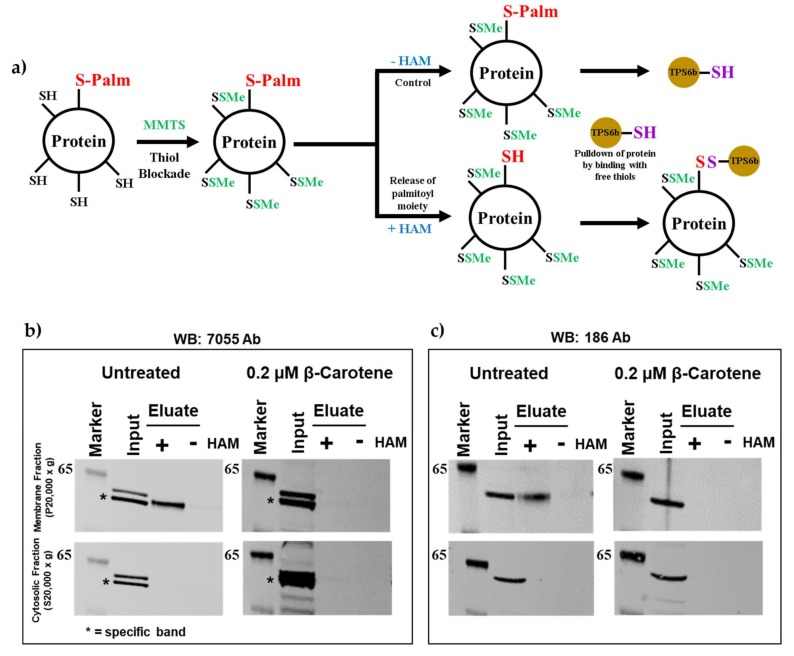
Detection of mBCO2 palmitoylation by acyl-RAC assay. (**a**) A schematic overview of the acyl-RAC method to detect the protein palmitoylation. Analysis of palmitoylation of mBCO2 from HEK293F-expressed lysates treated with 0.2 µM β-carotene in 0.01% OTG or 0.01% OTG vehicle control was performed by acyl-RAC assays. Samples were treated with a final concentration of 0.25 M hydroxylamine (HAM) or 0.25 M NaCl (control) and palmitoylated proteins were then enriched using beads (thiopropyl-sepharose 6b; TPS6b for acyl-RAC) and were eluted using 2.5% β-mercaptoethanol (BME) in SDS-PAGE sample buffer. Equal amounts (~20 µg) of total (indicated as “input”) and eluted protein from control (indicated as “−”) and HAM-treated (indicated as “+”) samples were separated by SDS-PAGE, followed by immunoblotting with primary rabbit polyclonal antibody to BCO2 (**b**) Analysis of palmitoylation of untagged mBCO2 from HEK293F-expressed lysates treated with β-carotene or vehicle control was performed by acyl-RAC assays. The presence of mBCO2 was probed by immunoblotting with rabbit polyclonal anti-human BCO2 (7055). Results are representative of three independent experiments. (**c**) Analysis of palmitoylation of untagged mBCO2 from HEK293F-expressed lysates treated with β-carotene or vehicle control was performed by acyl-RAC assays. The presence of mBCO2 was probed by immunoblotting with rabbit polyclonal anti-mouse BCO2 (186).

**Figure 2:**
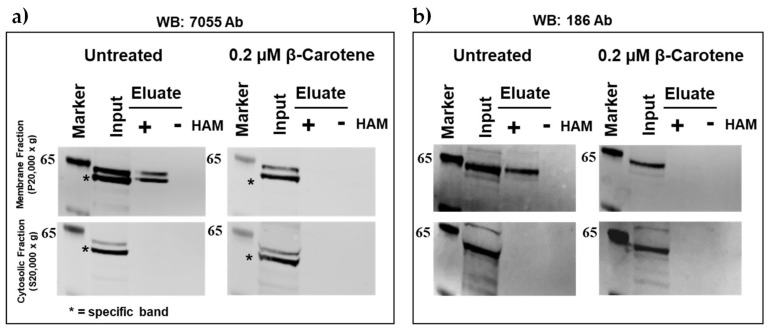
Detection of recombinant mBCO2/V5/Lumio protein palmitoylation by acyl-RAC assays. Analysis of palmitoylation of mBCO2/V5/Lumio protein from HEK293F-expressed lysates treated with 0.2 µM β-carotene in 0.01% OTG or 0.01% OTG vehicle control was performed by acyl-RAC assays. Samples were treated with a final concentration of 0.25 M hydroxylamine (HAM) or 0.25 M NaCl (control) and palmitoylated proteins were then captured using thiopropyl-sepharose beads and eluted using 2.5% BME in SDS-PAGE sample buffer. Equal amounts (~20 µg) of total (indicated as “input”) and eluted protein from control (indicated as “−”) and HAM-treated (indicated as “+”) samples were separated by SDS-PAGE, followed by immunoblotting with primary rabbit polyclonal antibody to BCO2. The top panel demonstrates results for membrane proteins recovered from the 20,000× *g* pellet and the bottom panel demonstrates results for the cytosolic proteins fraction (20,000× *g* supernatant). Results are representative of three independent experiments (raw data are submitted in Supplementary Materials). (**a**) Analysis of palmitoylation of mBCO2/V5/Lumio recombinant protein from HEK293F-expressed lysates treated with β-carotene or vehicle control. The presence of mBCO2 was probed by immunoblotting with rabbit polyclonal anti-human BCO2 (7055); (**b**) Analysis of palmitoylation of mBCO2/V5/Lumio recombinant protein from HEK293F-expressed lysates treated with β-carotene or vehicle control. The presence of mBCO2 was probed by immunoblotting with rabbit polyclonal anti-mBCO2 (186).

First, to establish the palmitoylation status of mBCO2 in eukaryotic cells we cloned mBCO2 cDNA into pVitro2 cloning vector and transfected in HEK293 suspension cells. Then cells were treated with either β-carotene in 0.01% n-Octyl-β-d-thioglucopyranoside (OTG) or 0.01% OTG vehicle control for 5 h. Additionally, we treated cells with 2.5 μM all-*trans*-retinol in ethanol, as the presence of all-*trans*-retinol in assays modulated RPE65 palmitoylation in our previous experiments [23]. We used the acyl-RAC method as shown in Figure 1a: In brief, following treatment of protein with the thiol-blocking agent S-methyl methanethiosulfonate (MMTS) to block free cysteine sulfhydryl (-SH) groups, hydroxylamine (HAM) was used to remove labile thioester-linked palmitoyl moieties in palmitoylated proteins, giving rise to free cysteine residues. These proteins with free cysteine residues were then captured and isolated using thiopropyl sepharose 6b (TPS6b) resin. Both membrane and cystosolic fractions of BCO2 were subjected to treatment with hydroxylamine (+HAM) and equal portions of the fractions were treated with NaCl (−HAM) which served as control. In the absence of β-carotene, BCO2 protein showed an intense protein band in the HAM-treated sample (Figure 1b,c; untreated panel), while there was no protein band in the control NaCl-treated sample, indicating S-palmitoylation of BCO2 protein. In contrast, when cells were pre-treated with 0.2 µM β-carotene, no protein band was detected in the HAM-treated sample (Figure 1b,c; β-carotene panel). Thus, our results demonstrate that mBCO2 is palmitoylated in the absence of β-carotene and that it loses palmitoylation when β-carotene is present (Figure 1b,c). RPE65 retinoid isomerase uses all-*trans*-retinyl esters as substrate and not carotenoids. To provide RPE65 with substrate we add all-*trans*-retinol to cells, relying on intracellular esterification [23]. We added the RPE65 pro-substrate all-*trans*-retinol to the mBCO2-expressing cells as a control condition to further determine if mBCO2 palmitoylation status depends specifically on the presence of its β-carotene substrate. We found that addition of all-*trans*-retinol did not affect the palmitoylation status of mBCO2 (Supplementary Figure S1). The majority of mBCO2 protein is present in the 20,000× *g* membrane pellet and only the membrane fraction of mBCO2 shows palmitoylation (Figure 1 and Figure 2).

Second, we cloned mBCO2 cDNA into pcDNA6.2c-Lumio-DEST cloning vector with C-terminal V5 and Lumio tags (mBCO2/V5/Lumio). We obtained the same palmitoylation profile in the presence and the absence of β-carotene confirming that the C-terminal tags do not affect palmitoylation status (Figure 2a,b). Mouse BCO2 was detected with custom made antibodies against mouse and human BCO2 peptides respectively (186 and 7055).

### 2.2. Mutations in C111 Change Palmitoylation Status of the Protein

**Figure 3:**
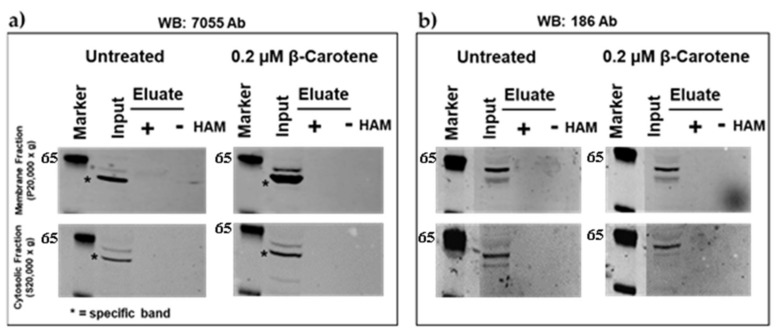
Detection of recombinant mouse C111S BCO2/V5/Lumio protein palmitoylation by acyl-RAC assays. Analysis of palmitoylation of mBCO2 from HEK293F-expressed lysates treated with 0.2 µM β-carotene in 0.01% OTG or 0.01% OTG vehicle control was performed by acyl-RAC assays. Top panel demonstrates results for membrane proteins recovered from 20,000× *g* pellet and bottom panel demonstrates results for cytosolic proteins fraction (20,000× *g* supernatant). Results are representative of three independent experiments (raw data are submitted in Supplementary Materials. (**a**) The presence of mutant mBCO2 was probed by immunoblotting with rabbit polyclonal anti-human BCO2 (7055); (**b**) The presence of mutant mBCO2 was probed by immunoblotting with rabbit polyclonal anti-mouse BCO2 (186).

To determine the palmitoylation site in mBCO2 we performed site-directed mutagenesis. We created the C111S cysteine mutant in tagged mBCO2 and additionally we investigated a mouse polymorphic variant P108SN190D BCO2 (BCO2SD), where one of these two amino acid changes modifies the conserved amino acid motif PDPCK to SDPCK) described previously [12]. We observed that palmitoylation was lost completely in the C111S mutant (Figure 3a,b) while it was preserved in the double mutant P108SN190D carrying the change of the first proline in the PDPCK motif to serine (Supplementary Figure S2.). The activity of the double mutant towards β-carotene was decreased by 40% in the bacterial *E. coli* system and its specificity was changed towards cleaving lycopene [12].

### 2.3. Cellular Localization of Mouse BCO2 with and without Substrate

**Figure 4:**
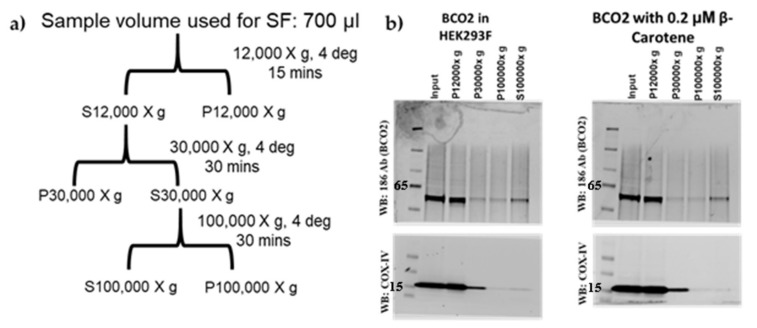
Sucrose Sub-cellular Fractionation 293F-overexpressed mBCO2. (**a**) Scheme of subcellular fractionation; (**b**) Immunoblotting of subcellular fractions of HEK293F cells transfected with mBCO2 with (right two panels) or without β-carotene (left two panels) with 186 (anti-BCO2) antibody (top panel) and mitochondrial marker COXIV antibody (bottom panel).

To determine if β-carotene-modulated palmitoylation influences membrane targeting, we performed sucrose gradient subcellular fractionation experiments of mBCO2-transfected HEK293F cells. We found that most of the recombinant protein is localized in the 12,000× *g* pellet and corresponds to the localization of mitochondrial cytochrome c oxidase subunit IV (COXIV), a mitochondrial cell marker (Figure 4a,b).

**Figure 5:**
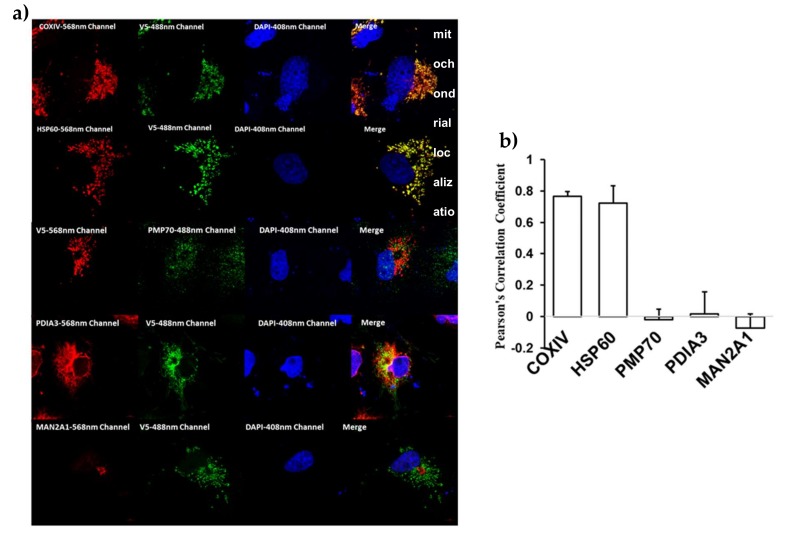
Characterization of BCO2 subcellular localization by immunofluorescence studies. (**a**) Subcellular localization of BCO2/V5/Lumio protein compared with different organelle markers. COS7 cells were transfected with BCO2/V5/Lumio protein, fixed 48 h following transfection, and immunostained for BCO2 using anti-V5 monoclonal mouse antibody (anti-V5-mAb) (or anti-V5 polyclonal rabbit antibody (Anti-V5-pAb) in the case of 70-kDa peroxisomal membrane protein (PMP70)), together with anti-COXIV mAb and anti-HSP60 mAb (mitochondria), anti-PMP70 mAb (peroxisomes), anti-protein disulfide-isomerase A3 (PDIA3) pAb (ER), or anti- alpha-mannosidase 2 (MAN2A1) pAb (Golgi), and analyzed by confocal microscopy. BCO2 protein shows an extensive colocalization with the mitochondrial marker proteins (COXIV or HSP60) in COS7 cells. (**b**) Pearson’s correlation coefficient for colocalization of BCO2/V5/Lumio protein with different organellar marker proteins. Results are presented as mean ± SD for 40–50 cells for each marker.

Thus, our initial data agrees with previously published results of murine BCO2 localization regardless of its palmitoylation status [24,25,26]. Because the 12,000× *g* pellet contains not only mitochondria but also peroxisomes and lysosomes, we therefore examined BCO2 localization by immunofluorescence microscopy using mouse BCO2 transfected COS7 cells with various organellar markers (for mitochondria, peroxisomes, endoplasmic reticulum (ER), and Golgi). Immunofluorescence results as shown in Figure 5a,b revealed the extensive mitochondrial colocalization with COXIV or heat-shock protein 60 (HSP60). We did not observe any colocalization of BCO2 with other organelles (Figure 5a,b) as indicated by the very low correlation coefficient score compared with mitochondria. Our data are in full agreement with previous results for human BCO2 [24].

We also performed immunofluorescence analyses on mBCO2-transfected COS7 cells treated with 0.2 µM β-carotene for 5 h. In addition to mitochondrial localization, we observed a spatial difference in the localization of BCO2 in cytoplasm and nucleus upon treatment with β-carotene compared to untreated BCO2 (Supplementary Figure S3). However, subcellular fractionation studies failed to detect an increased level of BCO2 protein localized in cytoplasm (Figure 4a). 

### 2.4. Phylogenetic Analysis of New Metazoan Carotenoid Oxygenase Subfamily without PDPCK Motif (ACOL)

**Figure 6:**
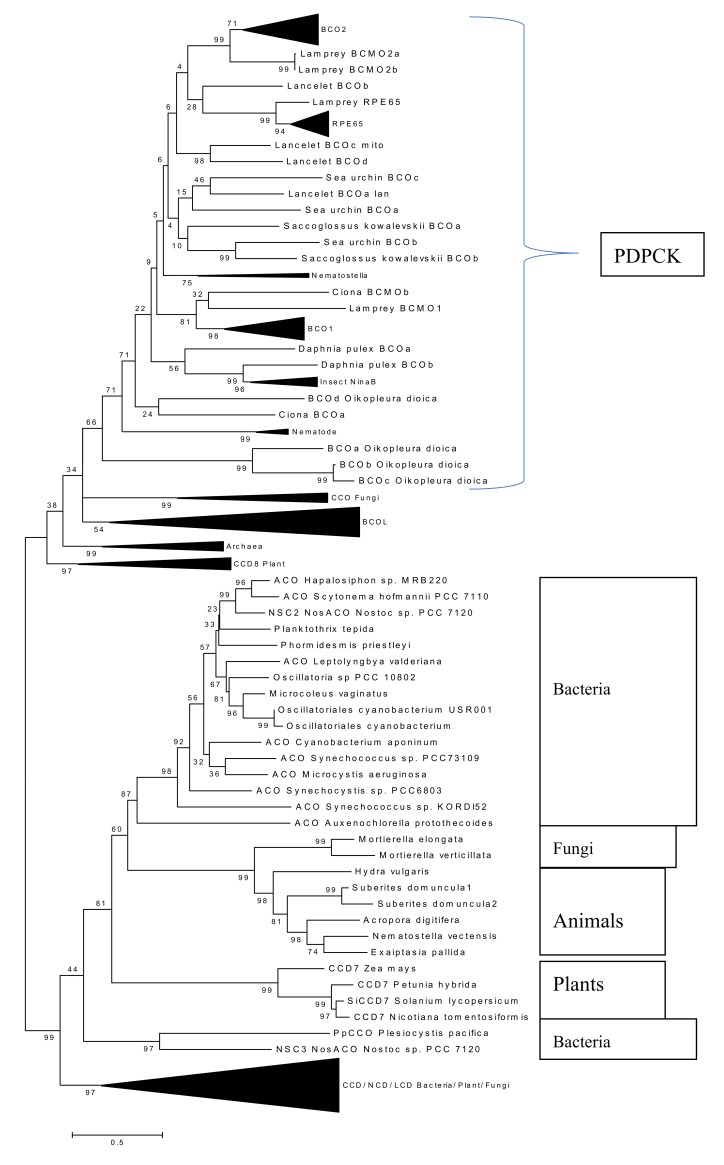
Molecular Phylogenetic analysis of the oxygenase superfamily by Maximum Likelihood (ML) method. The tree with the highest log likelihood (−10405.24) is shown. The percentage of trees in which the associated taxa clustered together (bootstrap support) is shown next to the branches. Initial tree(s) for the heuristic search were obtained automatically by applying Neighbor-Join and BioNJ algorithms to a matrix of pairwise distances estimated using a WAG (Whelan-and-Goldman) model, and then selecting the topology with superior log likelihood value. A discrete Gamma distribution was used to model evolutionary rate differences among sites (2 categories (+G, parameter = 1.1990)). The tree is drawn to scale, with branch lengths measured in the number of substitutions per site.

During a systematic analysis of the *Nematostella* (Cnidaria) genome and in tackling the problem of the unstable positioning of *Nematostella* CCOs on phylogenetic trees [12,27], we serendipitously uncovered a novel subfamily of animal ACO-like (ACOL) proteins. We found ACOL members in several cnidarian species and two putative orthologs in fungal species. Phylogenetic analysis of these ACOL proteins suggests that they form a well-supported clade (bootstrap support value = 99%) (Figure 6). All ACOL proteins contain the four canonical conserved histidines required for iron coordination in the active center but do not have the PDPCK motif. Their closest bacterial homologs are cyanobacterial ACO proteins. 

**Figure 7:**
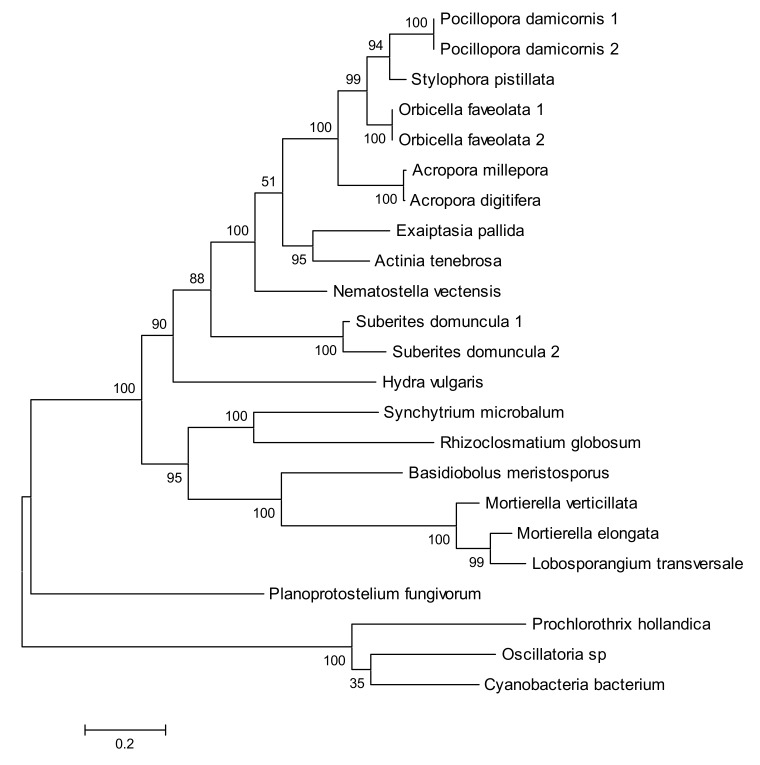
Molecular Phylogenetic analysis of the carotenoid oxygenase superfamily by Maximum Likelihood method. The tree with the highest log likelihood (−7455.23) is shown. The percentage of trees in which the associated taxa clustered together is shown next to the branches. Initial tree(s) for the heuristic search were obtained automatically by applying Neighbor-Join and BioNJ algorithms to a matrix of pairwise distances estimated using a JTT (Jones-Taylor-Thornton) model, and then selecting the topology with superior log likelihood value. A discrete Gamma distribution was used to model evolutionary rate differences among sites (2 categories (+G, parameter = 1.1990)). The tree is drawn to scale, with branch lengths measured in the number of substitutions per site.

We performed additional PSI-BLAST searches using various query sequences for database searches and found several putative ACOL orthologs in several fungal species and one amoebozoan species *Planoprotostelium fungivorum* (Figure 7). The putative amoebozoan ortholog is unlikely to be a bacterial contamination because of the presence of multiple introns. Thus, we conclude that the ACOL clade is an ancient eukaryotic protein family and the HGT hypothesis likely can be ruled out. We decided to check signs of functional changes in the course of the ACOL family evolution using a BLOSUM62-derived measure of amino acid differences (BDM, this BLOSUM62-derived measure is shown in the Supplementary Table S1, see Methods for details). We analyzed a long branch leading to the ACOL family between nodes 374 and 375 (Figure 6 and Supplementary Figure S4). We used branches 374–368, 373–374 and 375–376 as neighboring branches. BDM1 = 1656, R1 = 126; BDM2 = 1483, R2 = 112. The Fisher’s P value is equal to 0.55. Therefore BDM1 and BDM2 values are not different for the studied branches. This suggests that functional changes of ACOL compared to apocarotenoid oxygenases (ACO) are unlikely although results of the test should be treated with a caution (see the “Methods” section). Still, a plausible explanation is that the function of ACOL is likely to be the same or similar to cyanobacterial homologs consistent with lack of activity towards a full length carotenoids [28]. 

### 2.5. Absence of Palmitoylation in Carotenoid Oxygenase Metazoan Family Members without PDPCK Motif

**Figure 8:**
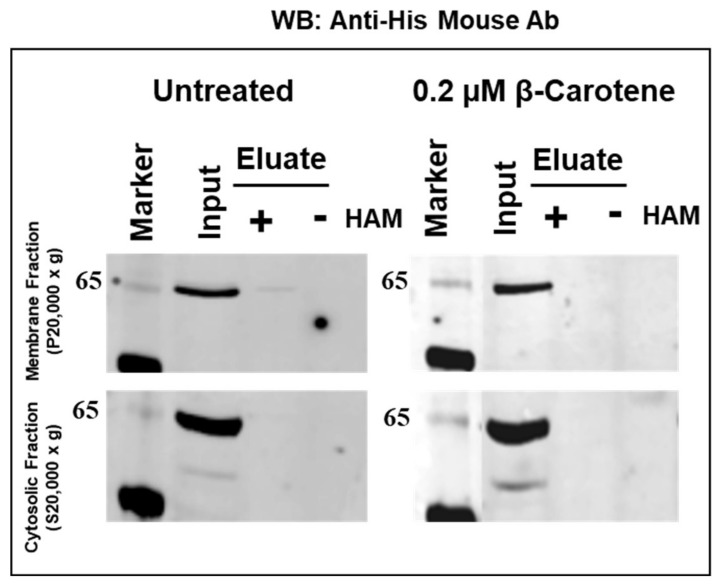
Analysis of palmitoylation of BCOLg in membrane (top panel) and cytosolic fraction (bottom panel) from HEK293F-expressed lysates treated with β-carotene as described in Materials and Methods. The presence of lancelet BCOLg was probed by immunoblotting with Anti-His Mouse monoclonal Roche antibody BMG-His-1 (1:1000) overnight at 4 °C.

To analyze the palmitoylation status of CCO members without the PDPCK motif, we first assayed the recently described BCOL subfamily member BCOLg (XP_002601288.1) [12]. We cloned BCOLg cDNA into the pCDNA3.1/His/V5/TOPO TA expression vector and transiently transfected it in HEK293F suspension cells for 43 h. Then cells were treated with β-carotene as described in Materials and Methods. We did not observe palmitoylation (Figure 8) and a significant fraction of protein was secreted out of the cells (Supplemental Figure S5). The BCOL proteins are predicted by TargetP to contain a signal peptide and, thus, are expected to be secreted [12].

**Figure 9:**
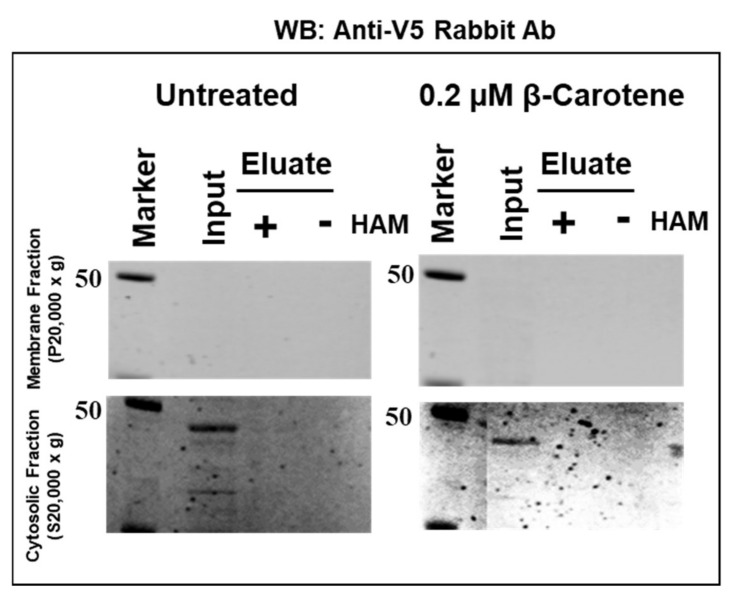
Analysis of palmitoylation of ACOLa in membrane (top panel) and cytosolic fraction (bottom panel) from HEK293F-expressed lysates treated with β-carotene as described in Materials and Methods. The presence of *Acropora* ACOLa was probed by immunoblotting with Anti-V5 mAb (1:1000).

Additionally, we cloned ACOLa (XM_029341311.1) cDNA, a new ACOL subfamily member, transfected it in HEK293F suspension cells for 43 h and then treated the transfected cells with β-carotene, as described previously. We did not detect palmitoylation and the protein was located only in the cytosolic fraction (Figure 9), confirming that proteins without the PDPCK motif lack palmitoylation. 

## 3. Discussion

Here we describe a further example of S-palmitoylation post-translational modification (PTM) in the metazoan CCO family. Additionally, we describe a new clade, ACOL, of the carotenoid oxygenase family that is predicted to be secreted. Finally, we develop a phylogenetic method for predicting changes in the functionality of enzymes in complex multifunctional families. 

Firstly, we report here the discovery of a new subfamily of eukaryotic CCOs, the ACOL family. This is in addition to the BCOL family we previously identified in animal genomes [12]. It is very tempting to speculate that these ACOL genes are the result of a horizontal gene transfer (HGT) event from cyanobacteria to cnidarian species. However the existence of fungal orthologs of ACOL would appear to rule out a recent HGT event: divergence of the fungal and animal clades is an ancient evolutionary event (>900 million years ago) shortly after the time of origin of eukaryotes themselves [29]. On the other hand, multiple separate HGT events could have occurred in the fungal species, in the sponges, and in the cnidarians harboring ACOLs. It is of interest to note that *Mortierella verticillata*, a fungus harboring an ACOL (Figure 6; along with a sister species *M. elongata*), has been shown to be a recipient of HGT [30], while sponges and cnidarians, and other early diverging metazoans, have been the subject of much interest in this area [31,32]. Two proteins from this subfamily were previously described in sponges (*Suberites domuncula*) and were found to have no β-carotene or lycopene cleavage activity in carotenoid producing *E. coli* [28]. 

We have previously shown that the CCO family member RPE65 retinoid isomerase is palmitoylated and that the extent of its palmitoylation is modulated by activity of lecithin:retinol acyltransferase (LRAT), the enzyme which provides RPE65′s all-*trans*-retinyl ester substrate [23]. We suggested that this apparent transferability of palmitoyl/acyl moieties between RPE65 and LRAT may play an important role in the efficiency of the vertebrate visual cycle [23]. We show here that mouse BCO2 is palmitoylated in the absence of substrate and not palmitoylated in the presence of β-carotene. This substrate- or activity-dependent palmitoylation may parallel the situation seen for RPE65. However, we did not find significant evidence that palmitoylation played a role in the mitochondrial membrane localization and trafficking of BCO2 [24,26]. There are various published reports suggesting versatile functions of protein palmitoylation besides subcellular localization. For example, palmitoylation has been shown to regulate protein stability, translocation to lipid rafts, trafficking, aggregation, and interaction with other proteins [33,34]. Accordingly, we speculate that palmitoylation may be involved in the catalytic function of the BCO2 enzyme. We observed that the addition of β-carotene and loss of palmitoylation leads to a more diffused pattern in the cells with partial nuclear and cytosolic involvement. We demonstrate that the C111 residue in the PDPCK motif is crucial for BCO2 palmitoylation, which is in agreement with recent RPE65 palmitoylation data [13,22,23]. On the other hand, metazoan carotenoid oxygenases that lack the PDPCK motif (such as BCOLg [12] and ACOLa) are not palmitoylated. This may be related to the particular protein subcellular localization of the different groups of CCOs (cytosolic or secreted). In future experiments, we will construct a chimeric protein adding the PDPCK motif to BCOLg and/or ACOLa in order to better understand the role of the PDPCK motif in governing CCO palmitoylation.

It has been reported that β-carotene is primarily localized to the nuclei of NCI-H69 small lung cancer cells and plays a protective role by reducing their proliferation rate [35]. Though we did not analyze the nuclear fraction (P300× *g*) in these experiments, it is possible that a small fraction of BCO2 protein is localized to the nucleus upon treatment with β-carotene. This possibility could illuminate the putative role of BCO2 protein in the nucleus, including the agonist function of BCO2 asymmetric cleavage products of β-carotene on RXR nuclear receptors [11]. This needs to be investigated in further detail.

These data provide further insight into the utilization of the palmitoylation PTM by a second mammalian BCO, and its lack of use by BCOLs and ACOLs. Expanding the universe of eukaryotic carotenoid oxygenases with identification of the ACOL group provides additional evidence for the functional flexibility of metazoan CCOs. 

## 4. Materials and Methods 

### 4.1. Generation of Expression Vectors and Site-Directed Mutagenesis

Mouse BCO2 cDNAs were subcloned into the bicistronic expression vector pVitro2 (InvivoGen, San Diego, CA, USA) and into the Gateway cloning vector pcDNA6.2c-Lumio-DEST vector (Thermo Fisher (Invitrogen), Carlsbad, CA, USA) to generate untagged or C-terminal V5/ lumio tagged versions of mouse BCO2, respectively. Lancelet *Branchiostoma floridae* BCOLg cDNA (XM_002601242.1) and cnidarian *Acropora digitifera* ACOL cDNA (XM_029341311.1) were subcloned into the expression vector pcDNA™3.1/V5-His TOPO. Site-directed mutagenesis of the mouse BCO2 open reading frane (ORF) was done using the QuikChange Lightning site-directed mutagenesis kit (Agilent, Santa Clara, CA, USA). All constructs and mutants were sequenced to verify the orientation and accuracy of the ORFs and/or the changes introduced.

### 4.2. Cell Culture

Human 293F FreeStyle (Thermo Fisher, Invitrogen) suspension cells were grown in serum-free FreeStyle 293 expression medium (Thermo Fisher, Invitrogen) and transfected according to the previously published protocol [18]. Briefly, a typical transfection experiment used 3 × 10^7^ cells in 28 mL of FreeStyle medium mixed with 2 mL of OptiMem-I reduced serum medium containing 40 μL 293fectin transfection reagent (Thermo Fisher, Invitrogen) and 20 μg of each expression plasmid under study. Cells were grown with shaking at 125 rpm on an orbital shaker platform in a 37 °C incubator with a humidified atmosphere of 8% CO_2_ in air for 48 h total. β-carotene was added in 0.01% OTG to the cells for 5 h (stock solution of β-carotene (200–400 µM) in hexane and 4% OTG mixed and dried under argon and resolubilized in 37 °C warm FreeStyle 293 expression medium). We carried out three independent transfections for the wild-type and mutant protein with or without β-carotene for acyl-RAC experiments. 

### 4.3. Sucrose Sub-Cellular Fractionation HEK293F-Overexpressed Mouse BCO2

HEK293F cells overexpressing mBCO2/no tags (BCO2 +/− carotene) were resuspended in 0.33 M sucrose in 10mM phosphate buffer pH 7.4, 1× Protease Inhibitor cocktail (Roche Diagnostics, Indianapolis, IN, USA). Cell lysis was performed using a N_2_ cavitation bomb (700 pounds per square inch (psi)). Nuclear debris was removed by centrifuging the cell lysate at 300× *g* for 5 min and the supernatant transferred to a fresh tube (volume = 2 mL). The supernatant (700 µL) was fractionated following the scheme in Figure 4a.

### 4.4. Antibodies

Rabbit polyclonal antibody 186 was custom-made against the mouse BCO2 multiple antigenic peptide (MAP)-SKFLQSDTYKANSAG peptide and 7055 Rabbit polyclonal antibody was produced by co-immunization of the two human BCO2 MAP-SHENLHQEDLEKEGGIE and MAP-QDNGRTLEVYQLQNLRKAG peptides.

### 4.5. Determination of BCO2 Palmitoylation by Acyl-RAC Method

Palmitoylation of the proteins was assayed by acyl-resin assisted capture (Acyl-RAC), as described previously with minor modifications [23,36]. Briefly, HEK293F cells overexpressing protein were washed with ice-cold 1× PBS (10 mM phosphate-buffered saline) and then resuspended in lysis buffer (50 mM HEPES pH 7.4, 150 mM NaCl, 5 mM EDTA, 1% glycerol and 1× complete protease inhibitor cocktail). Cells were disrupted using N_2_ cavitation under high pressure (700 psi) and the lysates were centrifuged at 300× *g* for 10 min at 4 °C to remove cell debris and nuclei. The post-nuclear supernatant was then centrifuged at 20,000× *g* for 30 min at 4 °C and the resulting membrane pellet was suspended in lysis buffer containing 0.1% Triton X-100. We used 500 µg of membrane and cytosolic fraction of HEK293F cells for palmitoylation detection analysis S-methyl methanethiosulfonate (MMTS) was used to block free sulfhydryls (-SH) of cysteine residues: an equal volume of 2× blocking buffer (0.1 M HEPES, 1 mM EDTA, 2.5% (*v*/*v*) SDS and 0.5% (*v*/*v*) MMTS; Sigma, St. Louis, MO, USA) was added to the membrane and cytosolic protein fraction and incubated for 15 min at 40 °C with frequent vortexing. Subsequently, three volumes of ice-cold 100% acetone was added to the blocking protein mixture and incubated for 30 min at −20 °C, and then centrifuged at 10,000× *g* for 10 min at 4 °C to pellet the precipitated proteins. The pellet was washed three times in 1 mL of ice-cold 70% (*v*/*v*) acetone and resuspended in buffer A (0.1 M HEPES, 5 mM EDTA, 1% SDS (*v*/*v*)). A fraction of the resuspended pellet was saved as the input. The remaining resuspended pellet was treated with hydroxylamine (HAM; Sigma) to cleave and release the palmitoyl moiety attached via thioester bond, and the proteins bearing free cysteine residues were captured by thiopropyl sepharose beads (Sigma). Briefly, ~ 200 µg of sample was treated with 2 M HAM together with the beads (previously activated with water for 15 min, washed and equilibrated with buffer A) to a final concentration of 0.25 M HAM and 10% (*w*/*v*) beads. As a negative control, 2 M NaCl (final concentration = 0.25 M NaCl) in 0.05 M HEPES buffer was used instead of HAM. Samples were then incubated at room temperature for 2 h with constant end-over-end mixing. After 2 h, samples were briefly centrifuged at 3000× *g* and the supernatants removed and retained as the “unbound” fraction. The remaining beads were washed three times with 1 mL buffer A. Subsequently, the proteins were eluted from the beads by incubating with 50 µL of Laemmli sample buffer (LSB:β-mercaptoethanol (BME):Buffer A, 0.9:0.1:3) for 15 min at room temperature and then 5 min at 95 °C. The eluted proteins were the “bound” fraction. Fractions were subjected to SDS-PAGE and analyzed by immunoblotting. Experiments were performed in triplicate.

### 4.6. Immunofluorescence, Confocal Microscopy and Analysis of BCO2 Colocalization in Different Subcellular Organelles (ER, Golgi, Mitochondria, and Peroxisomes)

COS7 cells seeded at 1 × 10^6^ cells/mL on 15 cm dish containing 1.8 cm poly-lysine coated coverslips (PLL; Neuvitro Corporation, Vancouver, WA, USA) were transfected with 20 µg of BCO2-Lumio tag pcDNA™3.1/V5-plasmid using Fugene^®^ 6 transfection reagent (Promega Corporation, Madison, WI, USA) in a 1:6 DNA:Fugene 6 ratio and grown in 37 °C incubator with 5% CO_2_ in air. At 48 h post transfection, coverslips with COS7 cells were washed three times with 10 mM PBS, pH 7.4 and fixed with fixative solution (4% paraformaldehyde in PBS pH 7.4; Electron Microscopy Sciences, Hatfield, PA, USA) for 15 min at room temperature. Fixed cells were washed with PBS and permeabilized with 0.1% Triton X-100 prepared in PBS followed by blocking with 1% BSA in PBS for overnight at 4 °C. Single and dual labeling of BCO2-Lumio tag (1:250 V5 mouse monoclonal (Sigma) and 1:500 V5 rabbit polyclonal (Cell Signaling Technology, Danvers, MA, USA)) and different organelles (ER, Golgi, mitochondria, and peroxisomes) were achieved by incubating cells with primary antibodies (ER, 1:250 PDIA3 rabbit polyclonal (Novus Biologicals, Littleton, CO, USA); Golgi, 1:200 Mannosidase II/ MAN2A1 rabbit polyclonal (Abcam, Cambridge, UK); Mitochondria, 1:250 COXIV rabbit monoclonal (Cell Signaling Technology) and 1:800 HSP60 rabbit monoclonal (Cell Signaling Technology); and Peroxisomes, 1:200 PMP70 mouse monoclonal (Cell Signaling Technology) in 0.1% bovine serum albumin (BSA) in PBS for 2 h at room temperature. Cells were then washed three times for 5 min each with 1× PBS containing 0.1% Triton X-100 and fluorescently labeled with 1:1000 donkey anti-mouse 488, donkey anti-rabbit 568 secondary antibodies (Invitrogen) for 2 h at room temperature. Cells were then incubated with 1 µg/µL 4′,6-diamidino-2-phenylindole (DAPI) solution (Sigma) prepared in DAPI assay buffer (0.01M Tris buffer, 0.1 M NaCl and 0.01 M EDTA) for 10 min at room temperature to stain the nuclei and washed three times for 5 min each with 1× PBS buffer. Coverslips were mounted on microscope slides using Prolong™ Glass Antifade Mountant (Thermo Fisher Scientific) and stored at 4 °C until imaged.

Imaging was performed using an inverted fluorescence microscope (LSM 700 confocal microscope; Zeiss, Oberkochen, Germany) equipped with four solid state lasers (405, 488, 555, and 639 nm). Photomicrographs were taken with 40× oil immersion lens/ 1.4-NA using Zeiss ZEN software. Each experiment was performed on a minimum of three biological replicates, with 5-10 fields of view, containing on average one to five cells per field of view, analyzed per organelle.

The level of colocalization between BCO2 and different organelles was performed using the “Colocalization” tab in the ZEN software (Carl Zeiss Microimaging, LLC). For colocalization analysis, all the imaging parameters such as objective lens, lamp power, wavelength collections, acquisition speed, and PMT setting were kept constant for all data acquisition. We used single label control samples (BCO2-/Lumio/V5 only and organelle-only control sample) to accurately set the scatterplot crosshairs for examination of the pixel distribution of single label-only population. The crosshair was set just above the population, and the threshold values for both the channels (488 nm and 568 nm) were noted. These threshold values for both the channels were used for double label experimental samples for measuring the colocalization coefficients. The equation for the colocalization coefficients in the ZEN software were derived from the Pearson’s correlation coefficient [37]. The calculation for colocalization coefficient were performed using specific region of interest (ROI), drawn using the Overlay tool in the software. The software automatically adjusts the scatterplot to show the pixel distribution for the selected ROI. Data were represented as mean ± SD.

### 4.7. Phylogenetic Analysis of Functionality in Carotenoid Oxygenase Family

Protein sequences were downloaded from the NCBI and ENSEMBL web sites. Similarity searches were performed using the non-redundant protein sequence database at the NCBI website and gapped BLASTP/PSI-BLAST programs. Multiple protein sequence alignments were constructed using the Muscle program and then adjusted by hand (Supplementary Data, details available upon request from Eugenia Poliakov). Phylogenetic trees based on multiple alignments of protein sequences were constructed using the maximum-likelihood method as implemented in MEGA program [38]. The “Find Best Model (ML)” function of the MEGA package was used to determine the appropriate substitution models for each dataset. The model with the lowest Bayesian Information Criterion (BIC) score was considered to best describe the substitution pattern for that dataset and was subsequently chosen for phylogenetic analysis. 

We decided to check signs of functional changes in the course of the ACOL family evolution using a BLOSUM62-derived measure of amino acid differences (BDM, this BLOSUM62-derived measure is shown in the Supplementary Table S1). A similar measure (radical-conservative replacement ratios) of selection process has been used for various applications [39,40,41]. However, this measure is known to have several shortcomings: it is affected by various mutational and compositional factors; in addition, small data volumes create problems for statistical tests [42,43]. In general, selectional inferences based on radical-conservative replacement ratios and similar measures should be treated with caution [42,43]. Our modified BLOSUM62-based test was implemented using the MEGA program as follows:

1) We reconstructed an ML tree for protein sequences (Figure 6 and Supplementary Figure S4) and reconstructed ancestral sequences using the ML method.
2) We chose several specific branches (pairs of ancestral sequences). We used branches leading to RPE65 and BCOL families (Figure 6) as control experiments [12,27]. We also analyzed the long branch leading to the ACOL family (Figure 6).
3) We counted the number of amino acid replacements (R1), we used sites where ancestral states were predicted with the probability > 0.5 to infer high-confidence ancestral states. We also estimated the difference for each amino acid replacement (using BDM values) and summed BDM values for all R1 (BDM1).
4) We estimated BDM and R variables for three or four neighboring branches (depending on the local tree topology) and sum results for those branches producing R2 and BDM2. We avoided terminal branches because all tested branches (leading to RPE65, BCOL, and ACOL protein families) were internal ones.
5) We compared BDM1, R1, BDM2 and R2 using the one-tail Fisher exact test that produced a probability value P. We did not use any bulk analysis of all branches thus we did not apply the Bonferroni correction for multiple tests. However, obtained P values should be treated with caution anyway. We performed two control experiments using RPE65 and BCOL protein families (Figure 6)

Control case #1: RPE65 Protein Family

We analyzed a long branch (231-232) leading to the RPE65 family between nodes 231 and 232 (Figure 6 and Supplementary Figure S4). We used branches 230–231, 230–224 and 232–253 as neighboring branches. BDM1 = 1714, R1 = 130; BDM2 = 719, R2 = 79. The Fisher’s P value is equal to 0.01. The low P value suggests that there have been dramatic changes in functional activity, this is indeed the case for RPE65 [27]. 

Control case #2: BCOL Protein Family

We analyzed a long branch leading to the BCOL family between nodes 322 and 323 (Figure 6 and Supplementary Figure S4). We used branches 311–322, 321–322 and 323–324 as neighboring branches. BDM1 = 719, R1 = 54; BDM2 = 453, R2 = 41. The Fisher’s P value is equal to 0.22. The insignificant P value does not suggest that there have been any changes in functional activity, this indeed may be the case for the BCOL family [12].

## Supplementary Materials

The following are available online, Figure S1: Detection of recombinant mouse BCO2 protein palmitoylation by acyl-RAC assays in the presence and absence all trans-retinol; Figure S2: Detection of double mutant mouse BCO2SD protein palmitoylation by acyl-RAC; Figure S3: Characterization of subcellular localization of mouse BCO2 upon treatment with β-carotene by immunofluorescence studies; Figure S4: Molecular Phylogenetic analysis of the oxygenase superfamily by Maximum Likelihood method; Figure S5: Presence of BCOLg protein in media of HEK293F transfected cells; Table S1: The BLOSUM63-derived measure of amino acid differences.
