# Supplementary material for: Palmitoylation of Metazoan Carotenoid Oxygenases
Source: Molecules. 2020 Apr 22;25(8):1942. doi: 10.3390/molecules25081942 (PMC7221588; doi:10.3390/molecules25081942)

Supplementary figures.

Supplementary Figure S1. **Detection of recombinant mouse BCO2 protein palmitoylation by acyl-RAC assays in the presence and absence all-*trans*-retinol.** Analysis of palmitoylation of mouse BCO2 from HEK293F-expressed lysates treated with 2.5 µM all-*trans*-retinol in ethanol was performed by acyl-RAC assays. Top panel demonstrates results for membrane proteins recovered from 20,000 x g pellet and bottom panel demonstrates results for cytosolic proteins fraction (20,000 x g supernatant). Results are representative of three independent experiments (raw data are submitted in Supplementary materials).

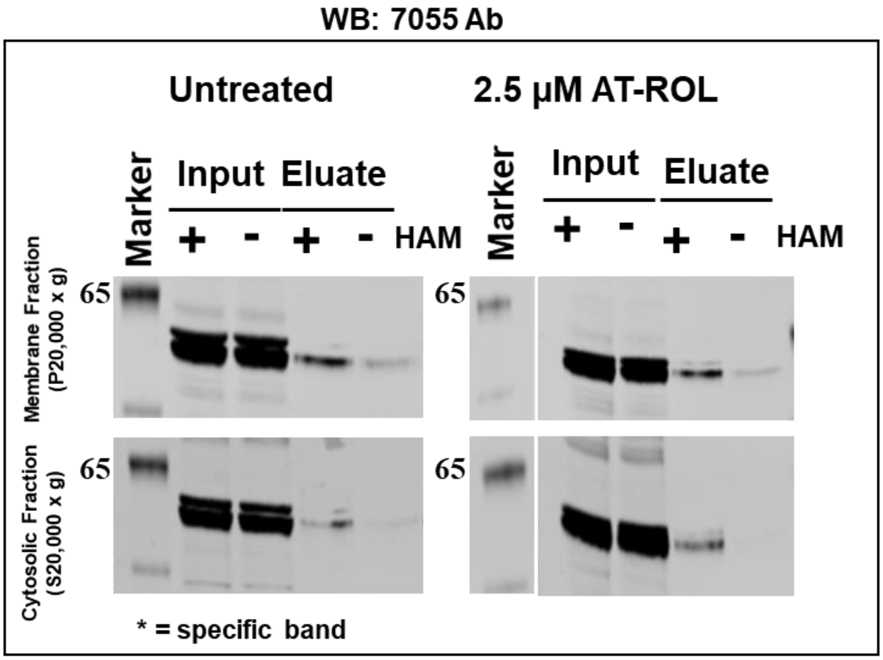

Supplementary Figure S2 **Detection of double mutant mouse BCO2SD protein palmitoylation by acyl-RAC assay.** The top panels demonstrate results for membrane proteins fractions recovered from 20,000 x g pellet and the bottom panels demonstrate results for the cytosolic proteins fractions (20,000 x g supernatant).(**a**) The presence of double mutant mouse BCO2 was probed by immunoblotting with rabbit polyclonal anti-human BCO2 (7055); (b)) The presence of double mutant mouse BCO2 was probed by immunoblotting with rabbit polyclonal anti-human BCO2 (186);

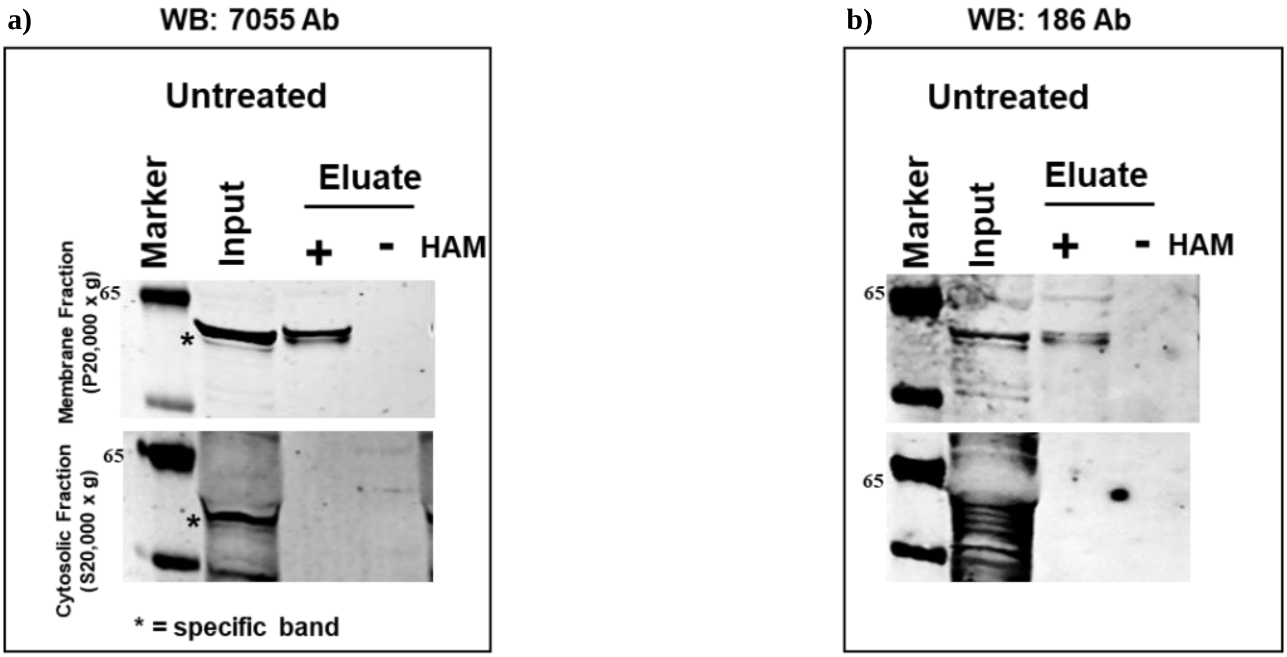

Supplementary Figure S3. **Characterization of subcellular localization of mouse BCO2 upon treatment with β-carotene by immunofluorescence studies.** COS7 cells were transfected with BCO2/V5/Lumio protein, fixed 48 hours following transfection, and immunostained for BCO2 using anti-V5 mAb together with mitochondrial anti-COXIV mAb (a) and anti-HSP60 mAb (b) and analyzed by confocal microscopy. In the case of β-carotene, cells were treated with 0.2 μM β-carotene after 24h post-transfection. Cells were fixed and immunostained following 5h treatment with β-carotene.

a)

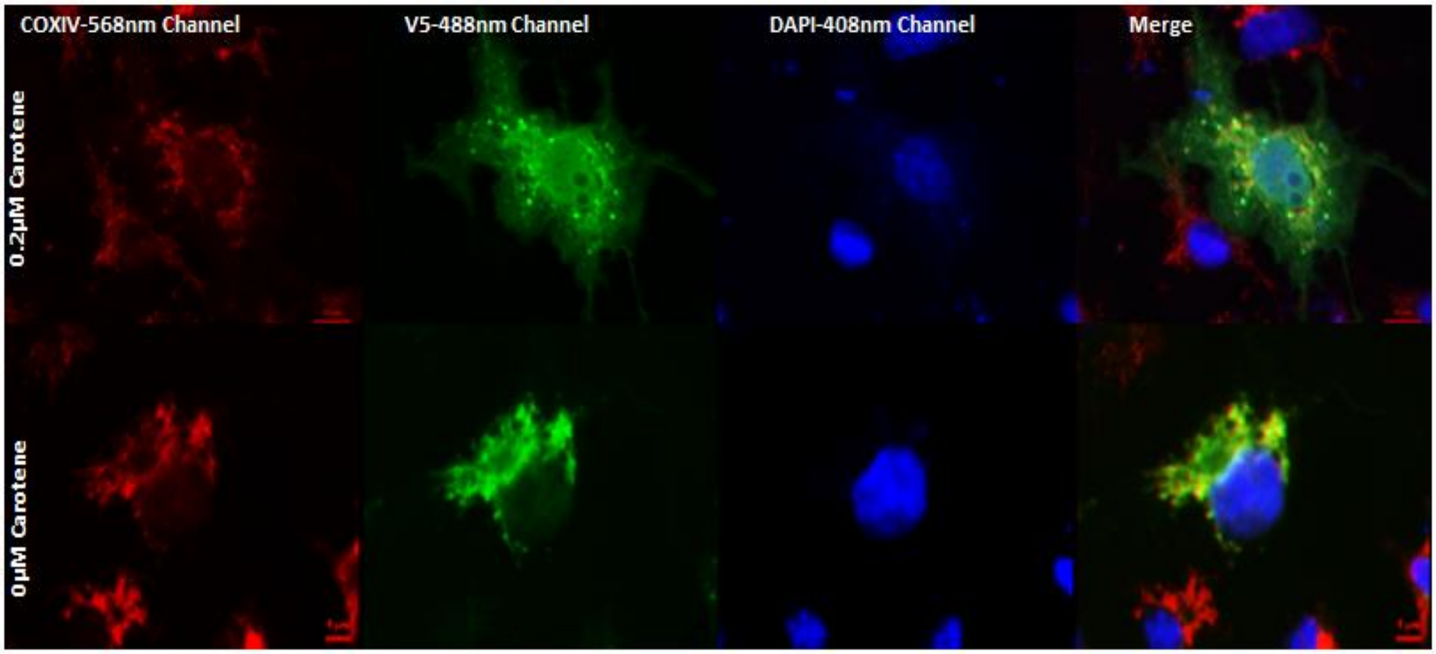

b)

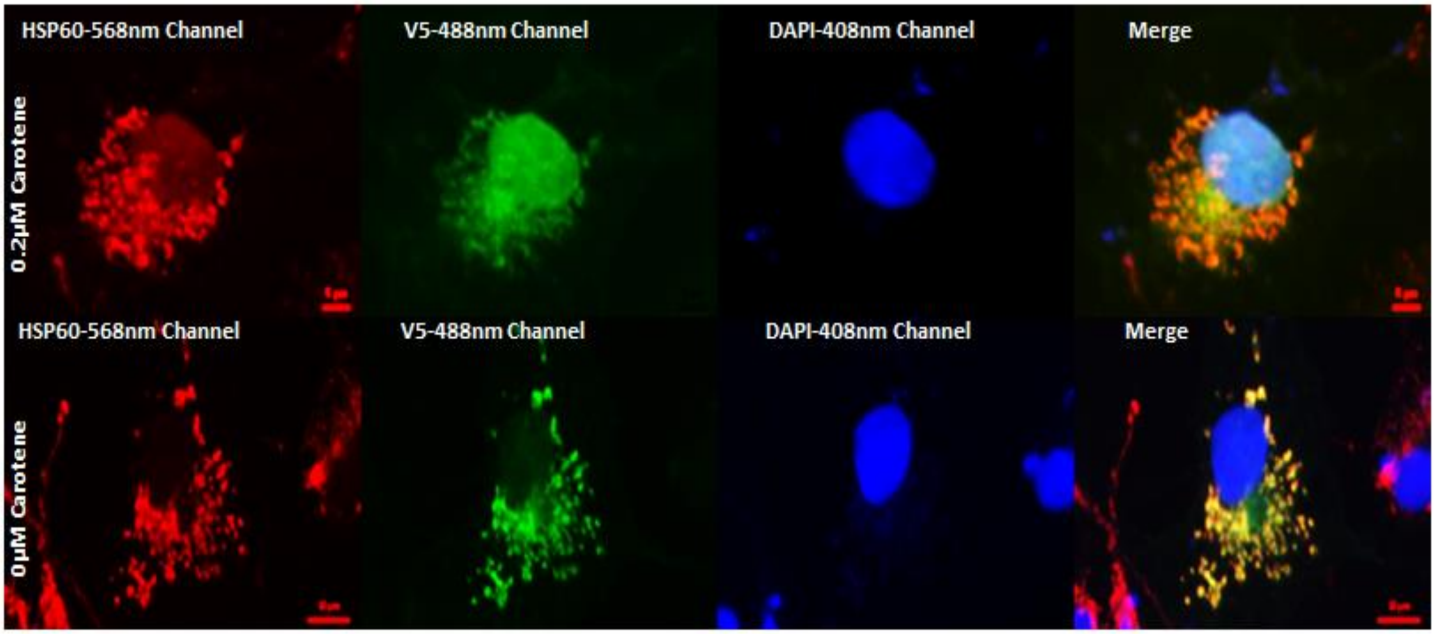

Supplementary Figure S4. **Molecular Phylogenetic analysis of the carotenoid cleavage oxygenase superfamily by Maximum Likelihood method.** The tree with the highest log likelihood (-10405.24) is shown. The label of nodes is shown next to the nodes. Initial tree(s) for the heuristic search were obtained automatically by applying Neighbor-Join and BioNJ algorithms to a matrix of pairwise distances estimated using a WAG model, and then selecting the topology with superior log likelihood value. A discrete Gamma distribution was used to model evolutionary rate differences among sites (2 categories (+*G*, parameter = 1.1990)). The tree is drawn to scale, with branch lengths measured in the number of substitutions per site. The figure should be zoomed (500%) for easy viewing.

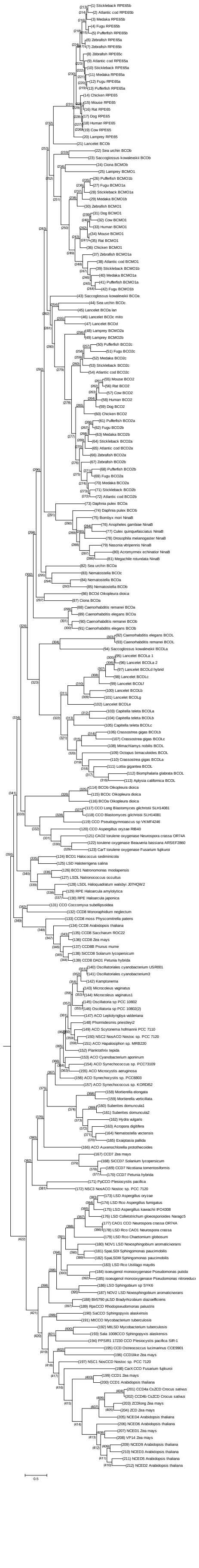
(1) Stickleback RPE65b
(2) Atlantic cod RPE65b
(3) Medaka RPE65b
(4) Fugu RPE65b
(5) Pufferfish RPE65b
(6) Zebrafish RPE65a
(7) Zebrafish RPE65b
(8) Zebrafish RPE65c
(9) Atlantic cod RPE65a
(10) Stickleback RPE65a
(11) Medaka RPE65a
(12) Fugu RPE65a
(13) Pufferfish RPE65a
(14) Chicken RPE65
(15) Mouse RPE65
(16) Rat RPE65
(17) Dog RPE65
(18) Human RPE65
(19) Cow RPE65
(20) Lamprey RPE65
(21) Lancelet BCOb
(22) Sea urchin BCOb
(23) Saccoglossus kowalevskii BCOb
(24) Ciona BCMOb
(25) Lamprey BCMO1
(26) Pufferfish BCMO1b
(27) Fugu BCMO1a
(28) Stickleback BCMO1a
(29) Medaka BCMO1b
(30) Zebrafish BCMO1
(31) Dog BCMO1
(32) Cow BCMO1
(33) Human BCMO1
(34) Mouse BCMO1
(35) Rat BCMO1
(36) Chicken BCMO1
(37) Zebrafish BCMO1a
(38) Atlantic cod BCMO1
(39) Stickleback BCMO1b
(40) Medaka BCMO1a
(41) Pufferfish BCMO1a
(42) Fugu BCMO1b
(43) Saccoglossus kowalevskii BCOa
(44) Sea urchin BCOc
(45) Lancelet BCOa lan
(46) Lancelet BCOc mito
(47) Lancelet BCOd
(48) Lamprey BCMO2a
(49) Lamprey BCMO2b
(50) Pufferfish BCO2c
(51) Fugu BCO2c
(52) Medaka BCO2c
(53) Stickleback BCO2c
(54) Atlantic cod BCO2c
(55) Mouse BCO2
(56) Rat BCO2
(57) Cow BCO2
(58) Human BCO2
(59) Dog BCO2
(60) Chicken BCO2
(61) Pufferfish BCO2a
(62) Fugu BCO2b
(63) Medaka BCO2b
(64) Stickleback BCO2a
(65) Atlantic cod BCO2a
(66) Zebrafish BCO2a
(67) Zebrafish BCO2b
(68) Pufferfish BCO2b
(69) Fugu BCO2a
(70) Medaka BCO2a
(71) Stickleback BCO2b
(72) Atlantic cod BCO2b
(73) Daphnia pulex BCOa
(74) Daphnia pulex BCOb
(75) Bombyx mori NinaB
(76) Anopheles gambiae NinaB
(77) Culex quinquefasciatus NinaB
(78) Drosophila melanogaster NinaB
(79) Nasonia vitripennis NinaB
(80) Acromyrmex echinatior NinaB
(81) Megachile rotundata NinaB
(82) Sea urchin BCOa
(83) Nematostella BCOc
(84) Nematostella BCOa
(85) Nematostella BCOb
(86) BCOd Oikopleura dioica
(87) Ciona BCOa
(88) Caenorhabditis remanei BCOa
(89) Caenorhabditis elegans BCOa
(90) Caenorhabditis remanei BCOb
(91) Caenorhabditis elegans BCOb
(92) Caenorhabditis elegans BCOL
(93) Caenorhabditis remanei BCOL
(94) Saccoglossus kowalevskii BCOLa
(95) Lancelet BCOLa 1
(96) Lancelet BCOLa 2
(97) Lancelet BCOLd hybrid
(98) Lancelet BCOLc
(99) Lancelet BCOLf
(100) Lancelet BCOLb
(101) Lancelet BCOLg
(102) Lancelet BCOLe
(103) Capitella teleta BCOLa
(104) Capitella teleta BCOLb
(105) Capitella teleta BCOLc
(106) Crassostrea gigas BCOLb
(107) Crassostrea gigas BCOLc
(108) Mimachlamys nobilis BCOL
(109) Octopus bimaculoides BCOL
(110) Crassostrea gigas BCOLa
(111) Lottia gigantea BCOL
(112) Biomphalaria glabrata BCOL
(113) Aplysia californica BCOL
(114) BCOb Oikopleura dioica
(115) BCOc Oikopleura dioica
(116) BCOa Oikopleura dioica
(117) CCO Long Blastomyces gilchristii SLH14081
(118) CCO Blastomyces gilchristii SLH14081
(119) CCO Pseudogymnoascus sp VKMF4246
(120) CCO Aspergillus oryzae RIB40
(121) CAO2 torulene oxygenase Neurospora crassa OR74A
(122) torulene oxygenase Beauveria bassiana ARSEF2860
(123) CarT torulene oxygenase Fusarium fujikuroi
(124) BCO1 Halococcus sediminicola
(125) LSD Haloterrigena salina
(126) BCO1 Natronomonas moolapensis
(127) LSDL Natronococcus occultus
(128) LSDL Haloquadratum walsbyi J07HQW2
(129) RPE Haloarcula amylolytica
(130) RPE Haloarcula japonica
(131) CCO Coccomyxa subellipsoidea
(132) CCD8 Monoraphidium neglectum
(133) CCD8 moss Physcomitrella patens
(134) CCD8 Arabidopsis thaliana
(135) CCD8 Saccharum ROC22
(136) CCD8 Zea mays
(137) CCD8B Prunus mume
(138) SlCCD8 Solanum lycopersicum
(139) CCD8 DAD1 Petunia hybrida
(140) Oscillatoriales cyanobacterium USR001
(141) Oscillatoriales cyanobacterium3
(142) Kamptonema
(143) Microcoleus vaginatus
(144) Microcoleus vaginatus1
(145) Oscillatoria sp PCC 10802
(146) Oscillatoria sp PCC 10802(2)
(147) ACO Leptolyngbya valderiana
(148) Phormidesmis priestleyi2
(149) ACO Scytonema hofmannii PCC 7110
(150) NSC2 NosACO Nostoc sp. PCC 7120
(151) ACO Hapalosiphon sp. MRB220
(152) Planktothrix tepida
(153) ACO Cyanobacterium aponinum
(154) ACO Synechococcus sp. PCC73109
(155) ACO Microcystis aeruginosa
(156) ACO Synechocystis sp. PCC6803
(157) ACO Synechococcus sp. KORDI52
(158) Mortierella elongata
(159) Mortierella verticillata
(160) Suberites domuncula1
(161) Suberites domuncula2
(162) Hydra vulgaris
(163) Acropora digitifera
(164) Nematostella vectensis
(165) Exaiptasia pallida
(166) ACO Auxenochlorella protothecoides
(167) CCD7 Zea mays
(168) SlCCD7 Solanum lycopersicum
(169) CCD7 Nicotiana tomentosiformis
(170) CCD7 Petunia hybrida
(171) PpCCO Plesiocystis pacifica
(172) NSC3 NosACO Nostoc sp. PCC 7120
(173) LSD Aspergillus oryzae
(174) LSD Rco Aspergillus fumigatus
(175) LSD Aspergillus kawachii IFO4308
(176) LSD Colletotrichum gloeosporioides Naragc5
(177) CAO1 CCO Neurospora crassa OR74A
(178) LSD Rco CAO1 Neurospora crassa
(179) LSD Rco Chaetomium globosum
(180) NOV1 LSD Novosphingobium aromaticivorans
(181) SpaLSDI Sphingomonas paucimobilis
(182) SpaLSDIII Sphingomonas paucimobilis
(183) LSD Rco Ustilago maydis
(184) isoeugenol monooxygenase Pseudomonas putida
(185) isoeugenol monooxygenase Pseudomonas nitroreduc
(186) LSD Sphingobium sp SYK6
(187) NOV2 LSD Novosphingobium aromaticivorans
(188) Blr5790 pLSD Bradyrhizobium diazoefficiens
(189) RpsCCO Rhodopseudomonas palustris
(190) SaCCO Sphingopyxis alaskensis
(191) MtCCO Mycobacterium tuberculosis
(192) MtLSD Mycobacterium tuberculosis
(193) Sala 1008CCO Sphingopyxis alaskensis
(194) PPSIR1 17230 CCO Plesiocystis pacifica SIR-1
(195) CCD Ostreococcus lucimarinus CCE9901
(196) CCD1like Zea mays
(197) NSC1 NosCCD Nostoc sp. PCC 7120
(198) CarX CCO Fusarium fujikuroi
(199) CCD1 Zea mays
(200) CCD1 Arabidopsis thaliana
(201) CCD4a CsZCD Crocus sativus
(202) CCD4b CsZCD Crocus sativus
(203) ZCDlong Zea mays
(204) ZCD Zea mays
(205) NCED4 Arabidopsis thaliana
(206) NCED6 Arabidopsis thaliana
(207) NCED1 Zea mays
(208) VP14 Zea mays
(209) NCED9 Arabidopsis thaliana
(210) NCED3 Arabidopsis thaliana
(211) NCED5 Arabidopsis thaliana
(212) NCED2 Arabidopsis thaliana
0.5

Supplemental Figure S5. **Presence of BCOLg protein in media of HEK293F transfected cells.** BCOLg was transfected into HEK293F cells (30 mL culture volume) for 48 hours and media was collected and concentrated to 250 μL on Amicon Ultra 30 K cut-off concentrators. HEK293F samples are from cells not transfected with DNA. Media was concentrated the same way. Proteins were immunoblotted with Anti-His6 Antibodies (Monoclonal mouse IgG1 antibodies Roche, 1:500)

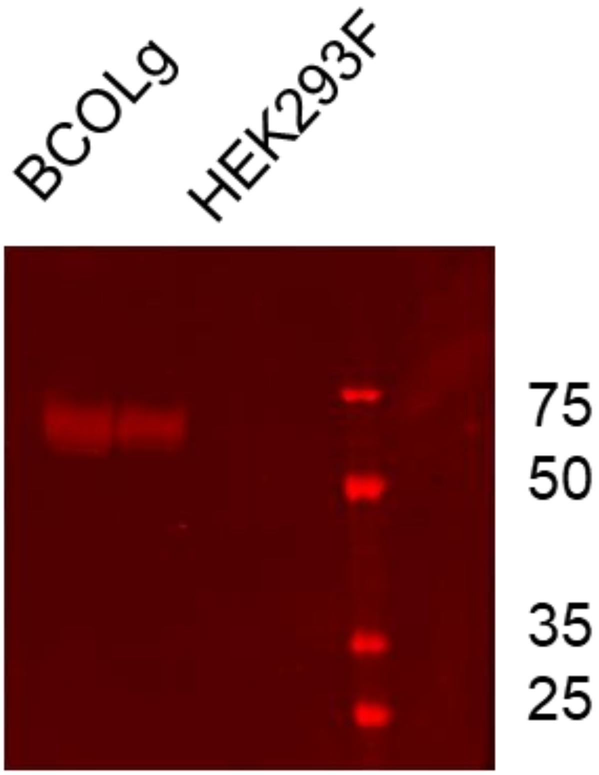

Supplementary Table S1. **The BLOSUM63-derived measure of amino acid differences.**

	A	C	D	E	F	G	H	I	K	L	M	N	P	Q	R	S	T	V	W	Y
A	4	-1	-2	-2	0	-1	-1	0	-2	-1	-1	-1	-1	-2	-1	1	0	-3	-2	0
C	-1	5	0	-2	-3	1	0	-2	0	-3	-2	2	-1	-3	-2	-1	-1	-3	-2	-3
D	-2	0	6	1	-3	0	0	0	1	-3	-3	0	-2	-3	-2	1	0	-4	-2	-3
E	-2	-2	1	6	-3	0	2	-1	-1	-3	-4	-1	-3	-3	-1	0	-1	-4	-3	-3
F	0	-3	-3	-3	9	-3	-4	-3	-3	-1	-1	-3	-1	-2	-3	-1	-1	-2	-2	-1
G	-1	1	0	0	-3	5	2	-2	0	-3	-2	1	0	-3	-1	0	-1	-2	-1	-2
H	-1	0	0	2	-4	2	5	-2	0	-3	-3	1	-2	-3	-1	0	-1	-3	-2	-2
I	0	-2	0	-1	-3	-2	-2	6	-2	-4	-4	-2	-3	-3	-2	0	-2	-2	-3	-3
K	-2	0	1	-1	-3	0	0	-2	8	-3	-3	-1	-2	-1	-2	-1	-2	-2	2	-3
L	-1	-3	-3	-3	-1	-3	-3	-4	-3	4	2	-3	1	0	-3	-2	-1	-3	-1	3
M	-1	-2	-3	-4	-1	-2	-3	-4	-3	2	4	-2	2	0	-3	-2	-1	-2	-1	1
N	-1	2	0	-1	-3	1	1	-2	-1	-3	-2	5	-1	-3	-1	0	-1	-3	-2	-2
P	-1	-1	-2	-3	-1	0	-2	-3	-2	1	2	-1	5	0	-2	-1	-1	-1	-1	1
Q	-2	-3	-3	-3	-2	-3	-3	-3	-1	0	0	-3	0	6	-4	-2	-2	1	3	-1
R	-1	-2	-2	-1	-3	-1	-1	-2	-2	-3	-3	-1	-2	-4	7	-1	-1	-4	-3	-2
S	1	-1	1	0	-1	0	0	0	-1	-2	-2	0	-1	-2	-1	4	1	-3	-2	-2
T	0	-1	0	-1	-1	-1	-1	-2	-2	-1	-1	-1	-1	-2	-1	1	5	-2	-2	0
V	-3	-3	-4	-4	-2	-2	-3	-2	-2	-3	-2	-3	-1	1	-4	-3	-2	11	2	-3
W	-2	-2	-2	-3	-2	-1	-2	-3	2	-1	-1	-2	-1	3	-3	-2	-2	2	7	-1
Y	0	-3	-3	-3	-1	-2	-2	-3	-3	3	1	-2	1	-1	-2	-2	0	-3	-1	4

Footnote: The BLOSUM63-derived measure of amino acid differences (BDM) was obtained using the equation BDM(i,j) = 12-BLOSUM62(i,j).

Raw western blots

Figure 1. Detection of mouse BCO2 palmitoylation by acyl-RAC assays.

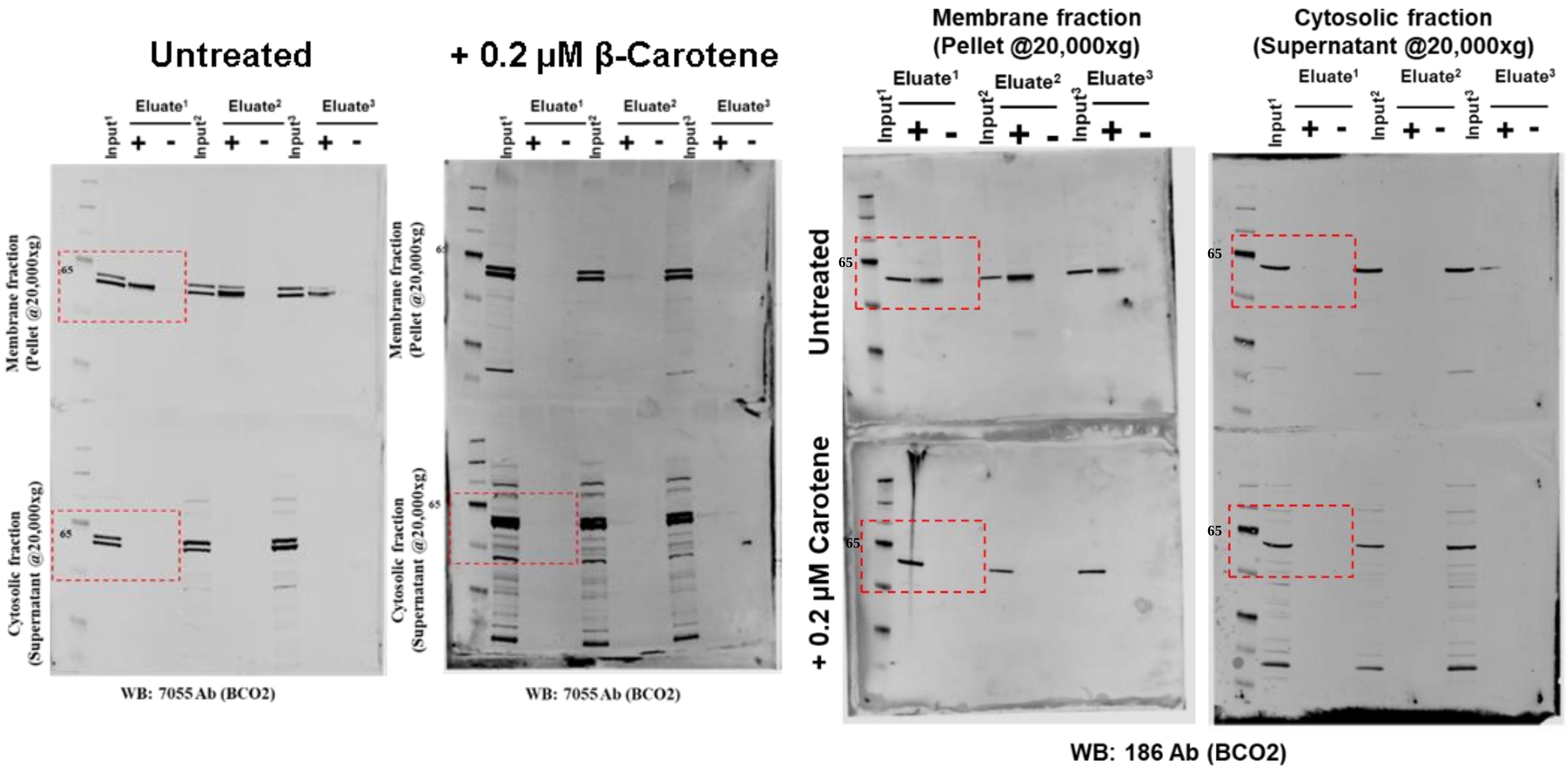

Figure 2. Detection of recombinant mouse BCO2/V5/Lumio protein palmitoylation by acyl-RAC assays.

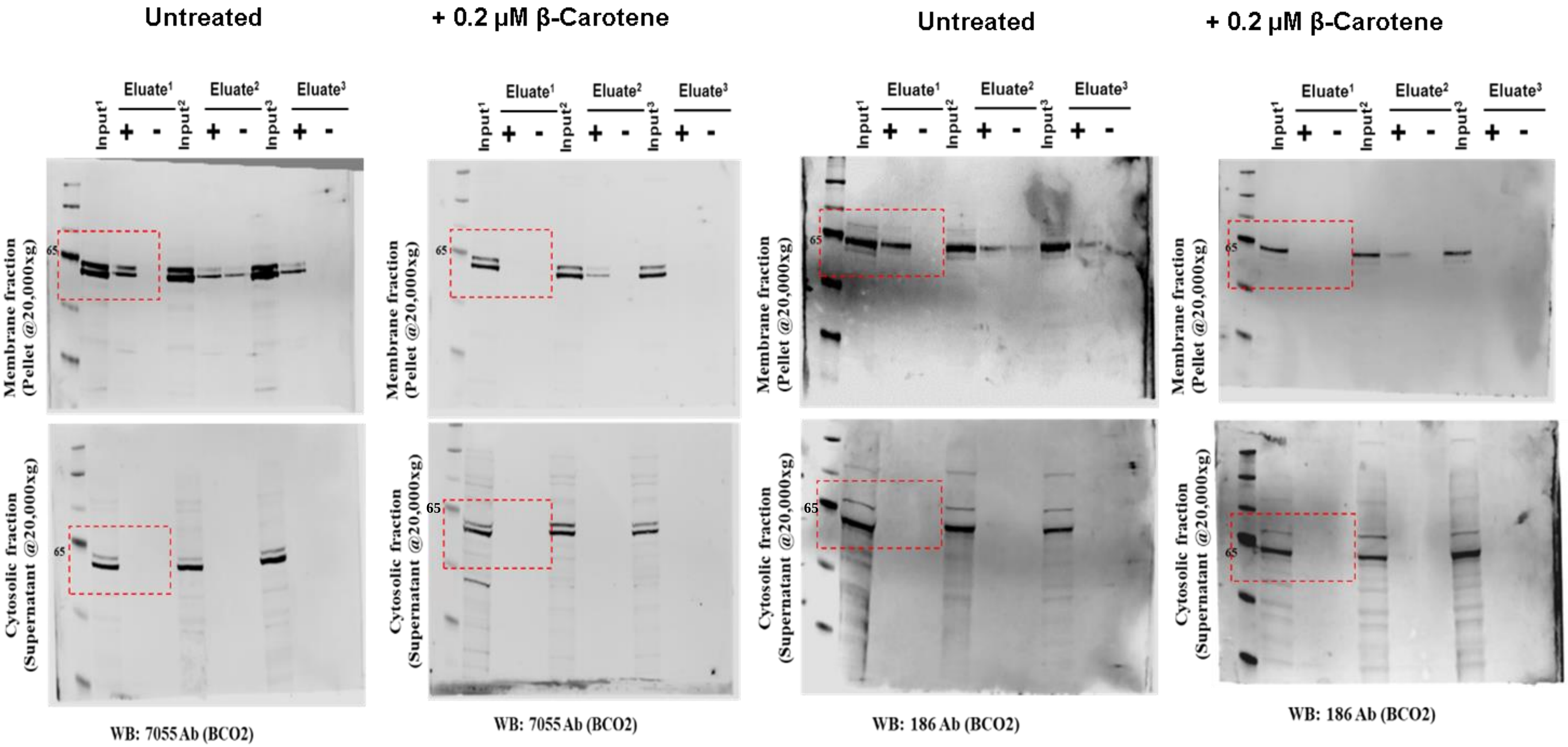

Figure 3. Detection of recombinant mouse C111S BCO2/V5/Lumio protein palmitoylation by acyl-RAC assays.

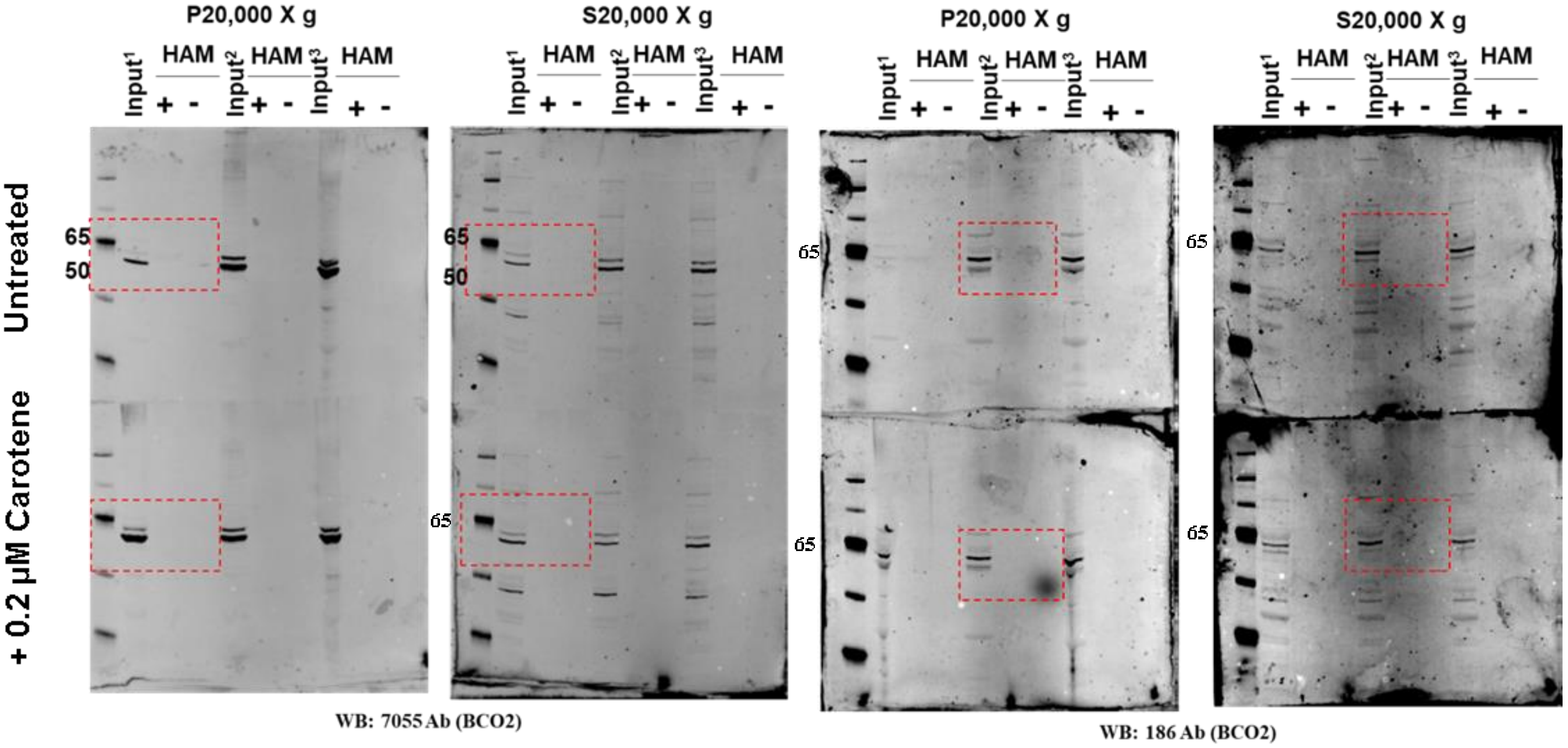

Figure 8. Analysis of palmitoylation of BCOLg in membrane (top panel) and cytosolic fraction (bottom panel) from HEK293F-expressed lysates treated with β-carotene as described in Materials and Methods.

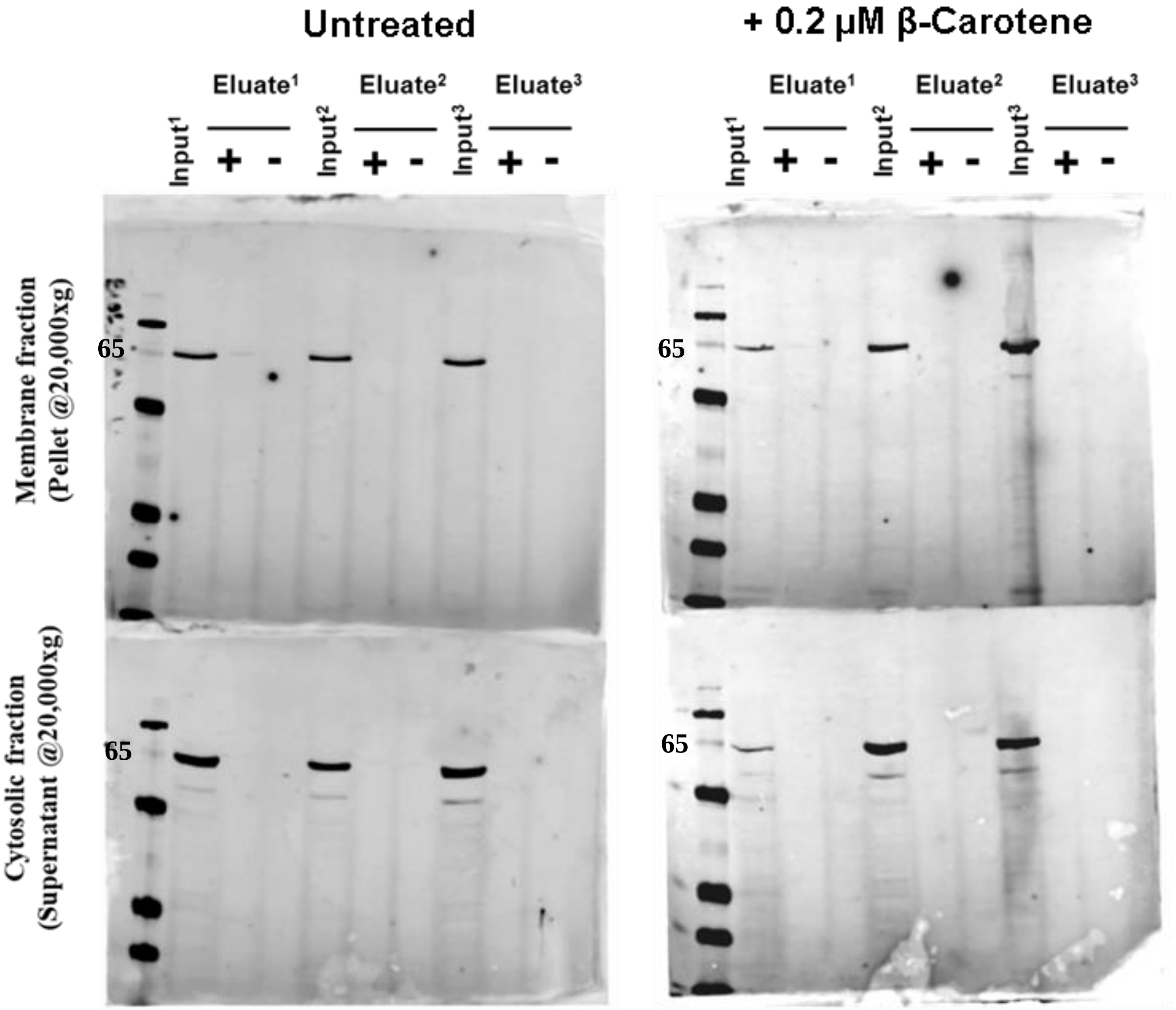

Figure 9. Analysis of palmitoylation of ACOLa in membrane (top panel) and cytosolic fraction (bottom panel) from HEK293F-expressed lysates treated with β-carotene as described in Materials and Methods.

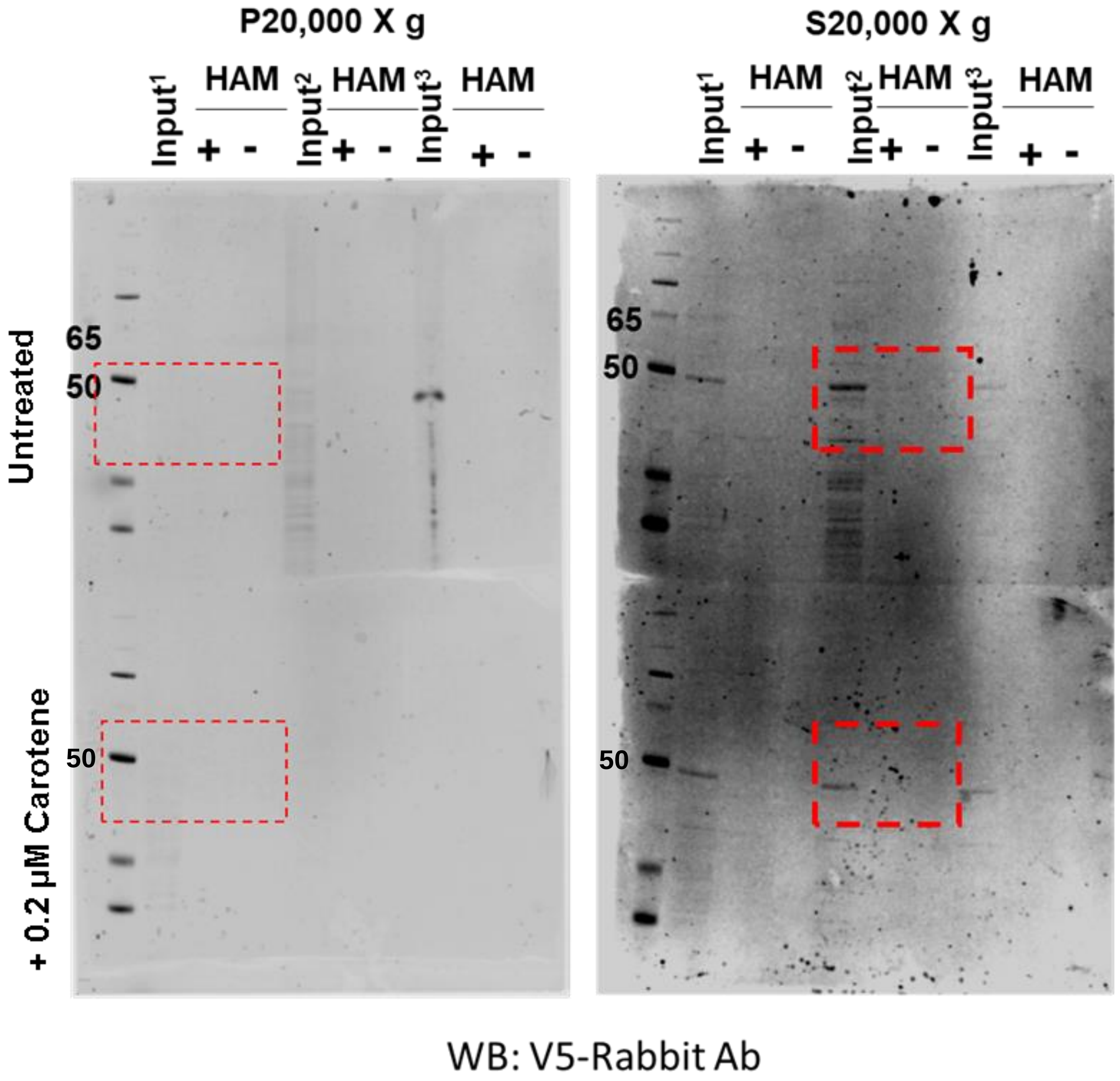

Figure 5. Characterization of BCO2 subcellular localization by immunofluorescence studies.

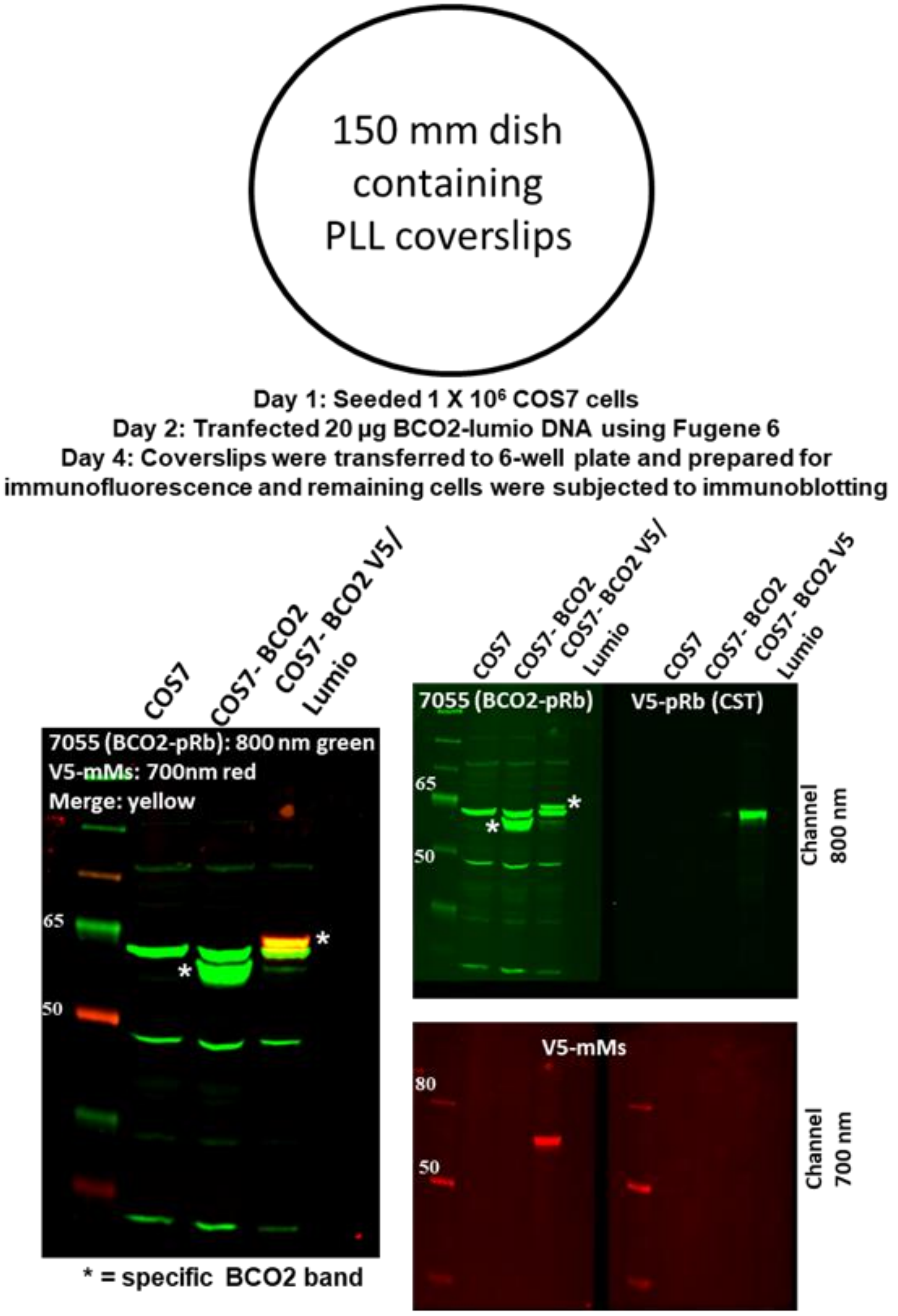

Immunofluorescence Images

COS7- No 1° and 2° Ab treatment

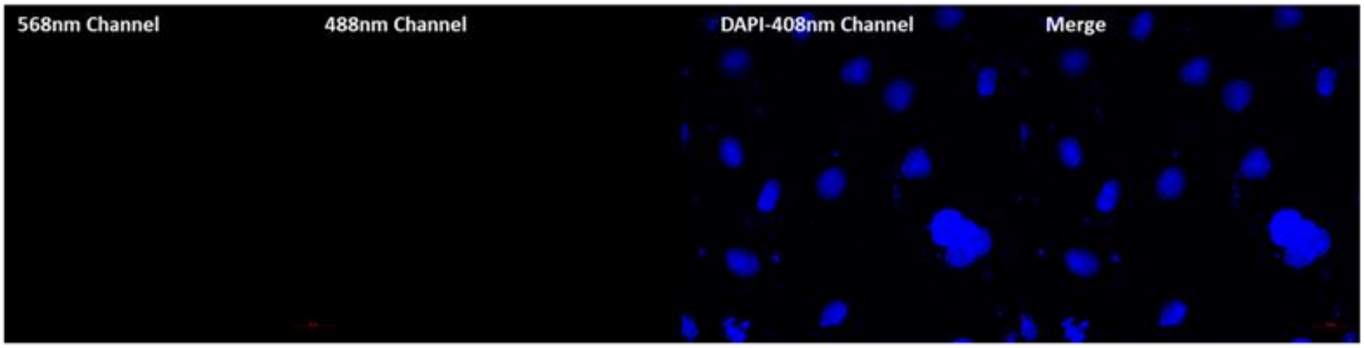

COS7- No 1° Ab treatment; 2° Ab Donkey anti Mouse 488

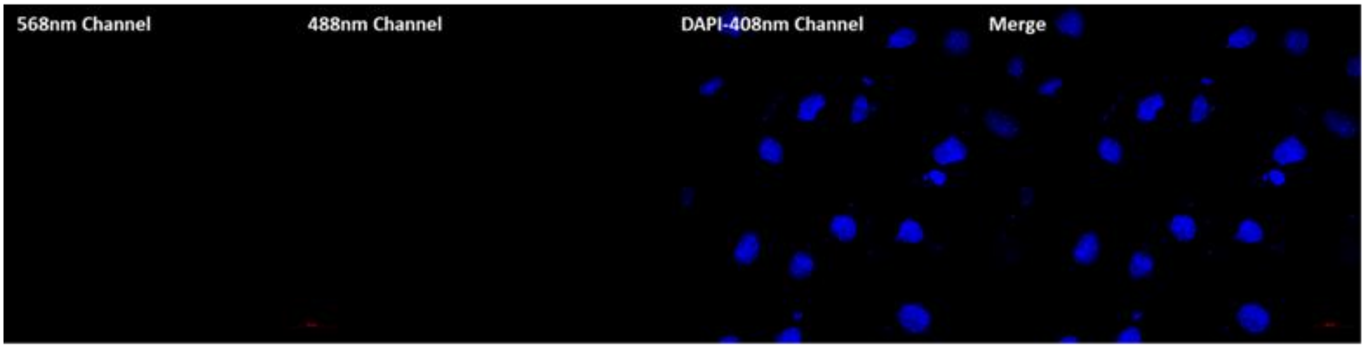

COS7- No 1° Ab treatment; 2° Ab Donkey anti Mouse 488 and Donkey anti Rabbit 568

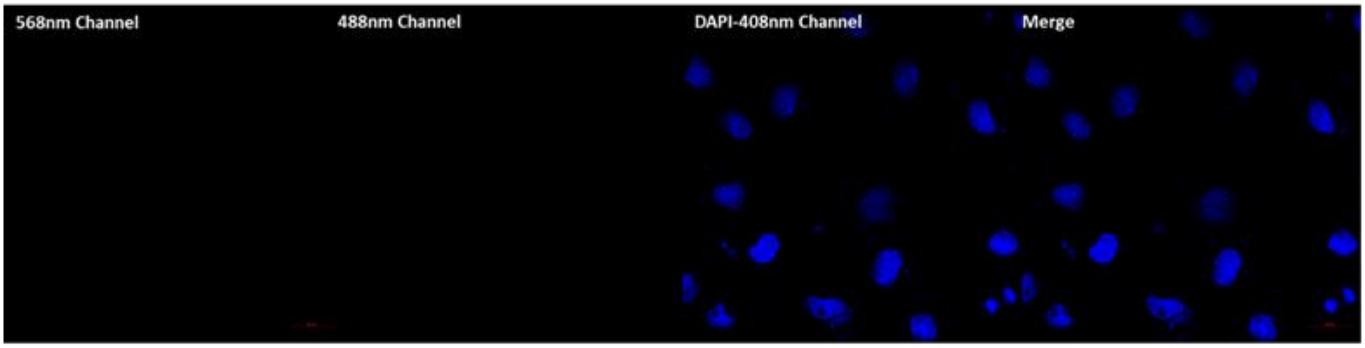

COS7- BCO2/V5/Lumio and COXIV (Mitochondrial marker protein) Colocalization

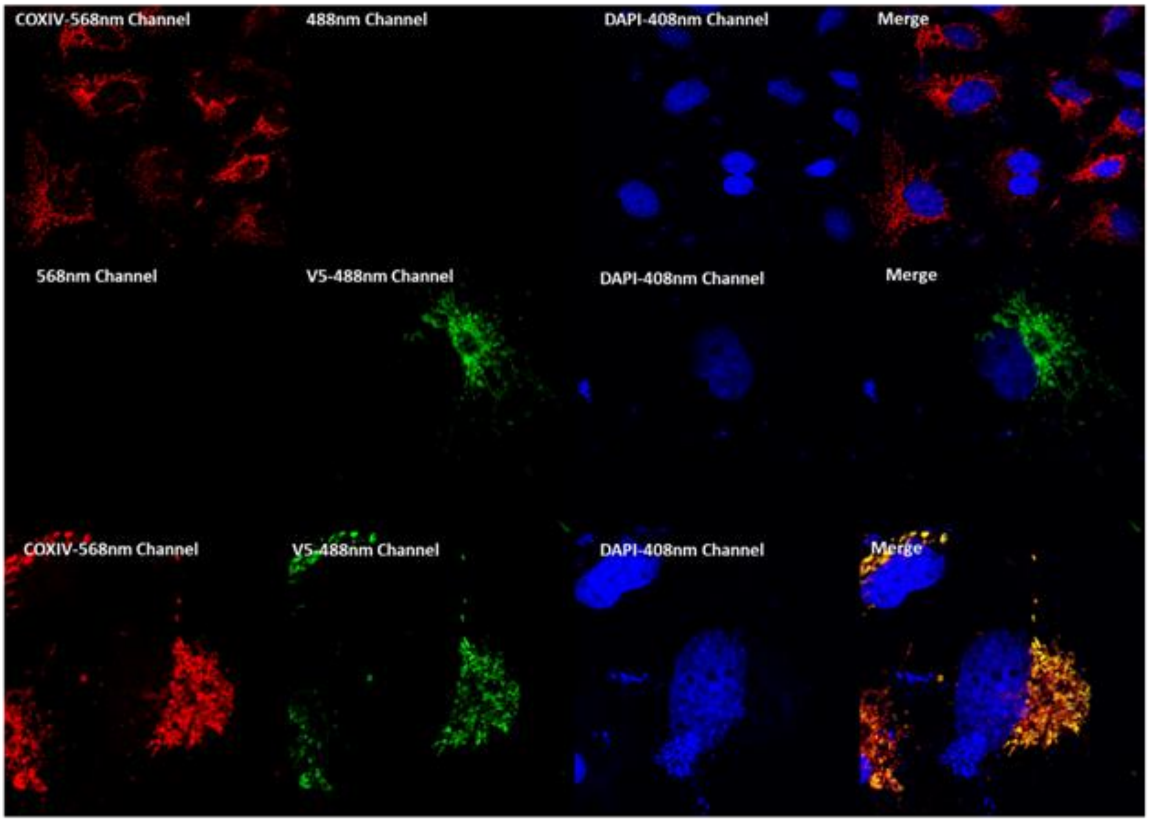

COS7- BCO2/V5/Lumio and HSP60 (Mitochondrial marker protein) Colocalization

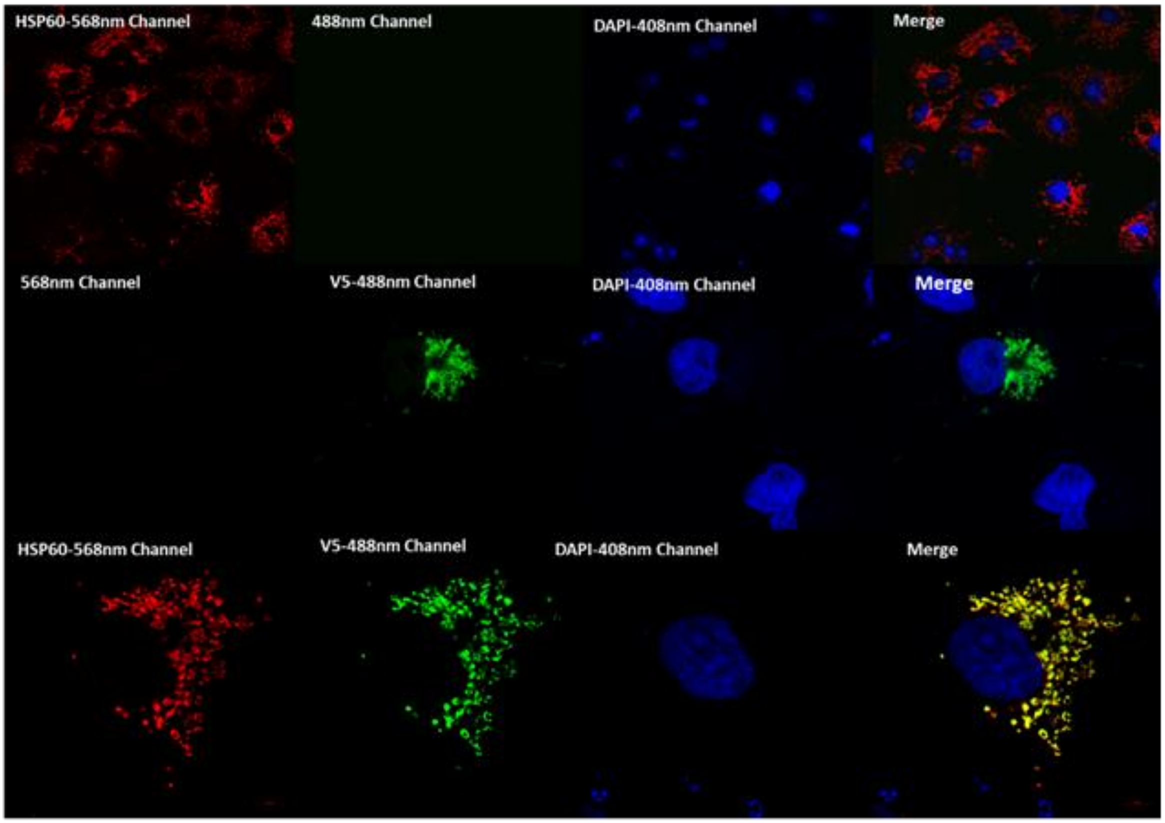

COS7- BCO2/V5/Lumio and PMP70 (Peroxisomal marker protein) Colocalization

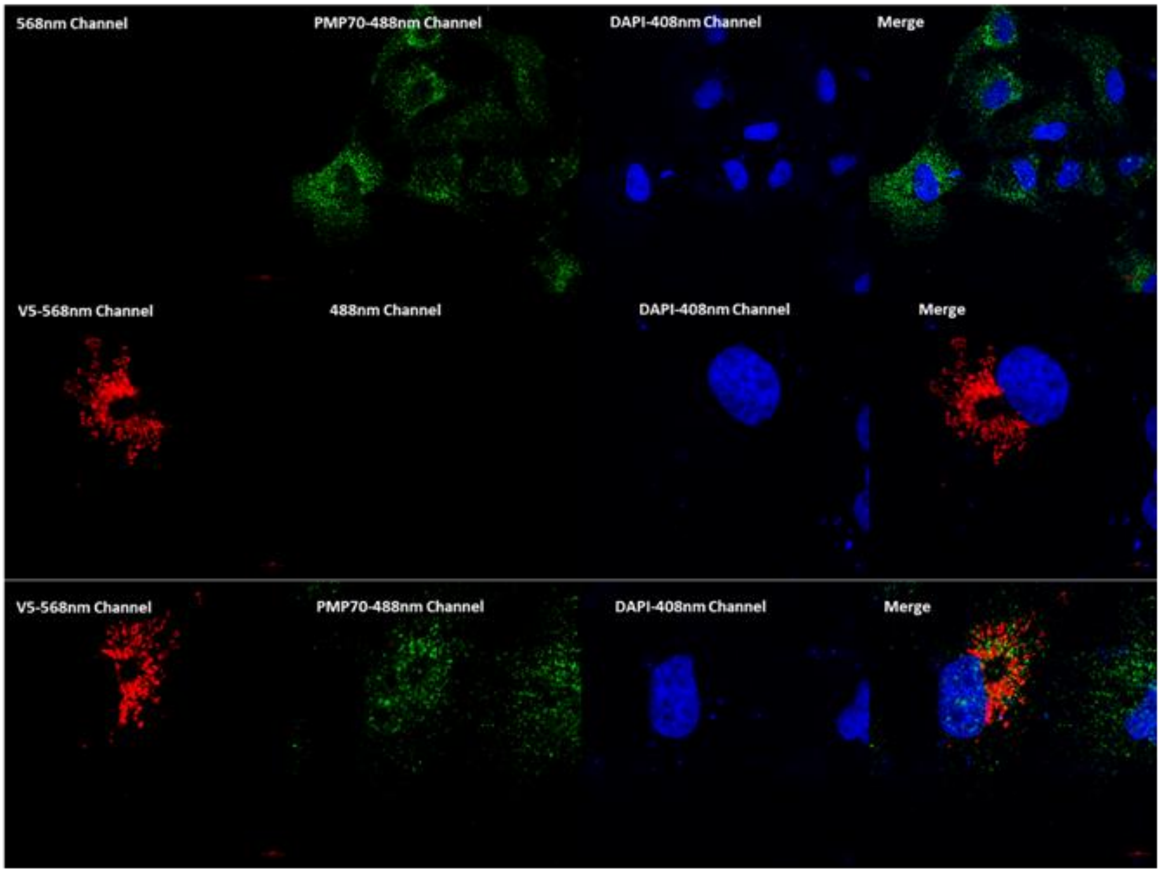

COS7- BCO2/V5/Lumio and PDIA3 (ER marker protein) Colocalization

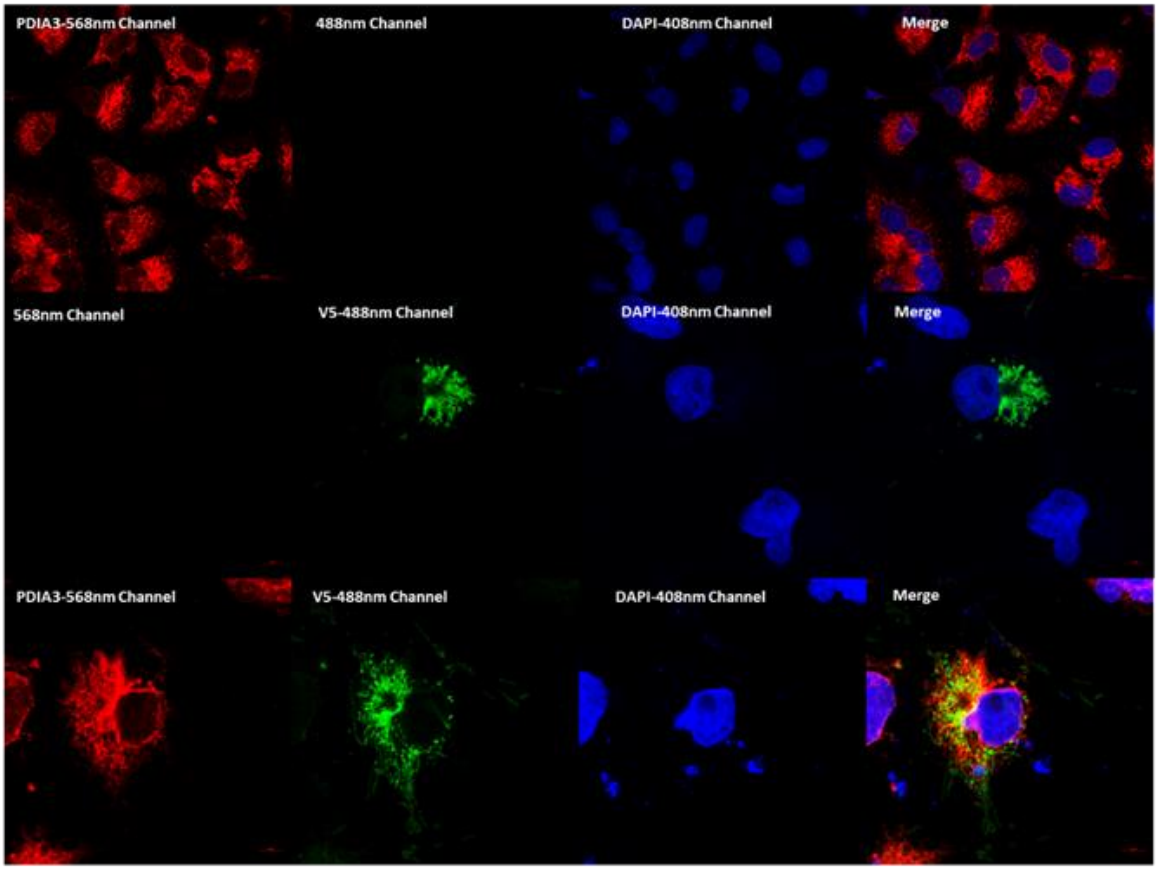

COS7- BCO2/V5/Lumio and MAN2A1 (Golgi marker protein) Colocalization

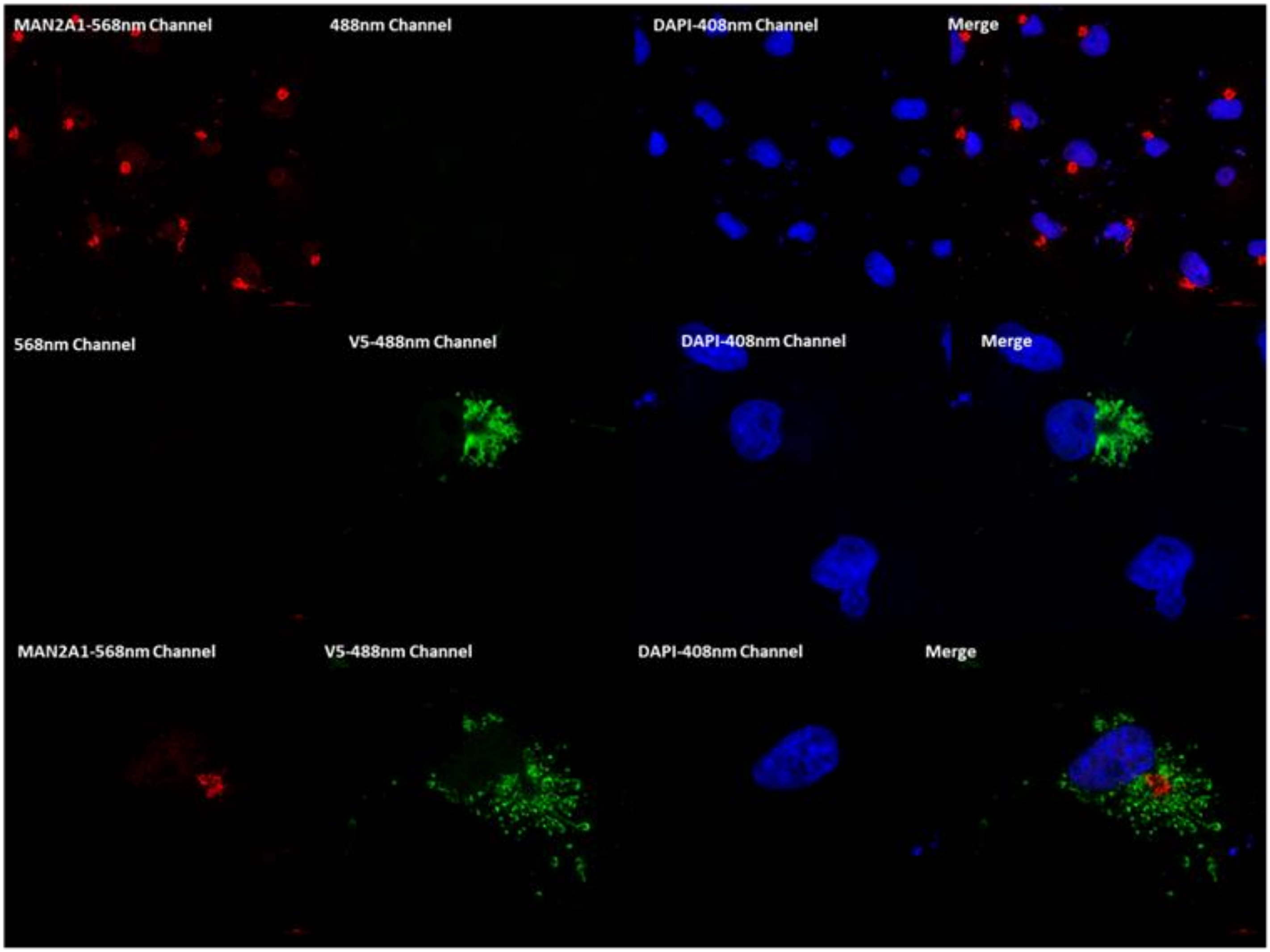

Supplementary Figure S1. **Detection of recombinant mouse BCO2 protein palmitoylation by acyl-RAC assays in the presence and absence all-*trans*-retinol.**

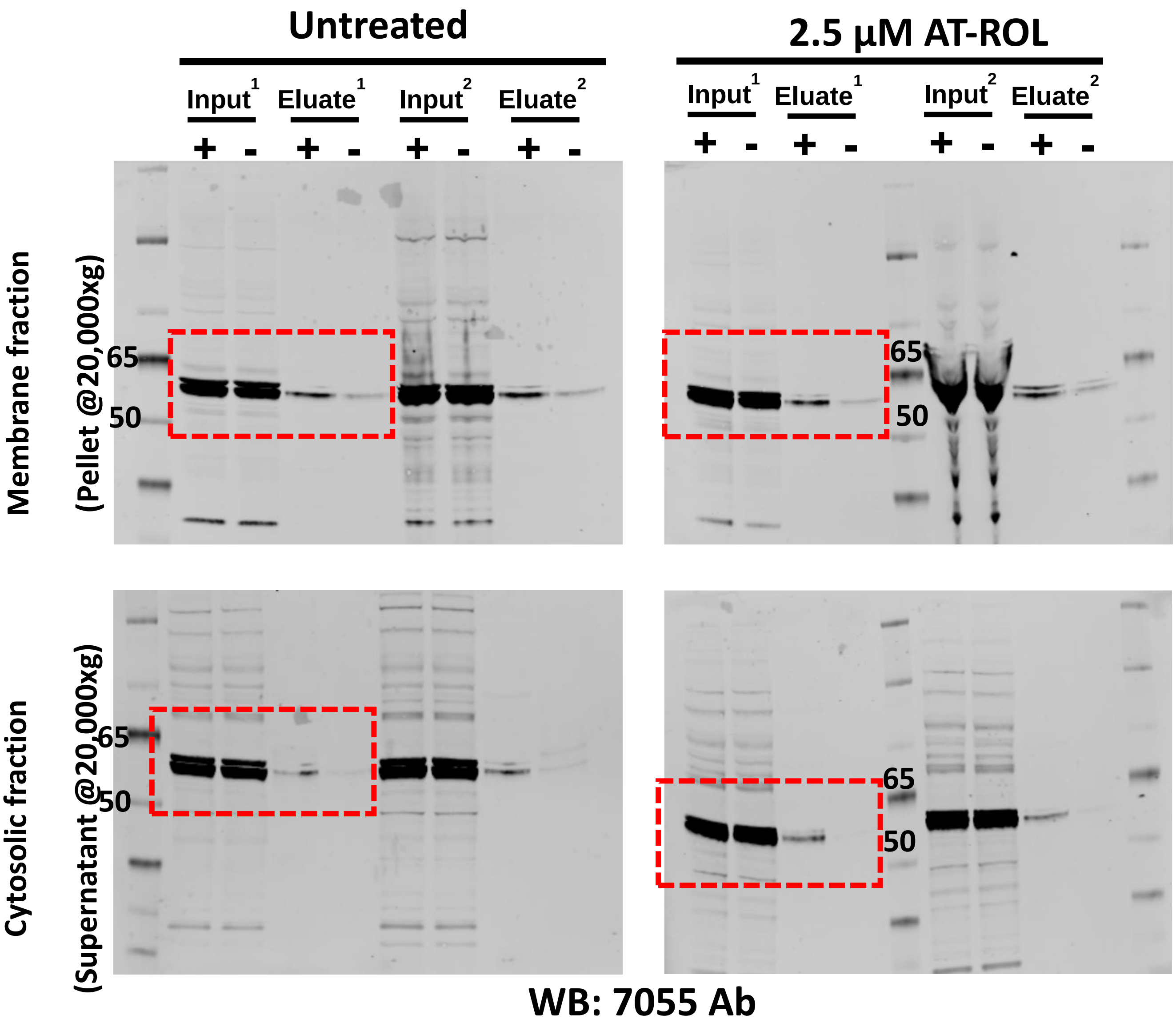

Supplement: Supplementary file 1 [file molecules-25-01942-s001.pdf]
